# An examination of protist diversity in serpentinization-hosted ecosystems of the Samail Ophiolite of Oman

**DOI:** 10.3389/fmicb.2023.1139333

**Published:** 2023-05-04

**Authors:** Alta E. G. Howells, Francesca De Martini, Gillian H. Gile, Everett L. Shock

**Affiliations:** ^1^School of Life Sciences, Arizona State University, Tempe, AZ, United States; ^2^Mesa Community College, Mesa, AZ, United States; ^3^Biodesign Center for Fundamental and Applied Microbiomics, Arizona State University, Tempe, AZ, United States; ^4^School of Molecular Sciences, Arizona State University, Tempe, AZ, United States; ^5^School of Earth and Space Exploration, Arizona State University, Tempe, AZ, United States

**Keywords:** protists, serpentinization, water-rock reaction, ecology, geochemistry

## Abstract

In the Samail Ophiolite of Oman, the geological process of serpentinization produces reduced, hydrogen rich, hyperalkaline (pH > 11) fluids. These fluids are generated through water reacting with ultramafic rock from the upper mantle in the subsurface. On Earth’s continents, serpentinized fluids can be expressed at the surface where they can mix with circumneutral surface water and subsequently generate a pH gradient (∼pH 8 to pH > 11) in addition to variations in other chemical parameters such as dissolved CO_2_, O_2_, and H_2_. Globally, archaeal and bacterial community diversity has been shown to reflect geochemical gradients established by the process of serpentinization. It is unknown if the same is true for microorganisms of the domain Eukarya (eukaryotes). In this study, using 18S rRNA gene amplicon sequencing, we explore the diversity of microbial eukaryotes called protists in sediments of serpentinized fluids in Oman. We demonstrate that protist community composition and diversity correlate significantly with variations in pH, with protist richness being significantly lower in sediments of hyperalkaline fluids. In addition to pH, the availability of CO_2_ to phototrophic protists, the composition of potential food sources (prokaryotes) for heterotrophic protists and the concentration of O_2_ for anaerobic protists are factors that likely shape overall protist community composition and diversity along the geochemical gradient. The taxonomy of the protist 18S rRNA gene sequences indicates the presence of protists that are involved in carbon cycling in serpentinized fluids of Oman. Therefore, as we evaluate the applicability of serpentinization for carbon sequestration, the presence and diversity of protists should be considered.

## 1. Introduction

Serpentinization, a subsurface geological process, involves reactions between water and ultramafic rock that produce reduced, hyperalkaline (>pH 11), H_2_-rich fluids (McCollom, 2007; Leong and Shock, 2020). Serpentinized fluids can supply H_2_ to archaea and bacteria whose metabolisms involve redox reactions including aerobic hydrogen oxidation, sulfate reduction and methanogenesis (McCollom, 2007; Canovas et al., 2017) and many serpentinization-hosted ecosystems are populated by these organisms (Morrill et al., 2014; Miller et al., 2016; Brazelton et al., 2017; Crespo-Medina et al., 2017; Rempfert et al., 2017; Twing et al., 2017; Fones et al., 2019; Howells et al., 2022). During serpentinization, water alters minerals in ultramafic rocks and the resulting serpentinized fluids can mix with surface water to generate geochemical gradients (Leong et al., 2021).

Archaeal and bacterial community compositions vary along geochemical gradients in serpentinization-hosted ecosystems. For example, a study of an active submarine serpentinization site called Prony Hydrothermal Field, located at the southern end of New Caledonia in the Bay of Prony, demonstrated that community compositions differ by system type, submarine or intertidal (Frouin et al., 2018). At The Cedars, an active terrestrial serpentinization site located in California, community composition correlates with fluid types which include deep fluids that have reacted with ultramafic rock and marine sedimentary layers, shallow groundwater that has reacted only with the ultramafic rock, or fluids that are mixed to some extent with meteoric water (Suzuki et al., 2013). In the active terrestrial serpentinization system of the Samail Ophiolite in Oman archaeal and bacterial community compositions are related to the type of ultramafic rock with which the water interacts, and the degree to which the serpentinized fluid mixes with surrounding surface water or shallow subsurface water (Miller et al., 2016; Rempfert et al., 2017; Fones et al., 2019, 2021; Kraus et al., 2021; Howells et al., 2022). While drivers of archaeal and bacterial community composition in serpentinization-hosted ecosystems are being documented, it is unknown whether the factors that drive bacterial and archaeal diversity also drive microbial eukaryote diversity in these systems.

**FIGURE 1:**
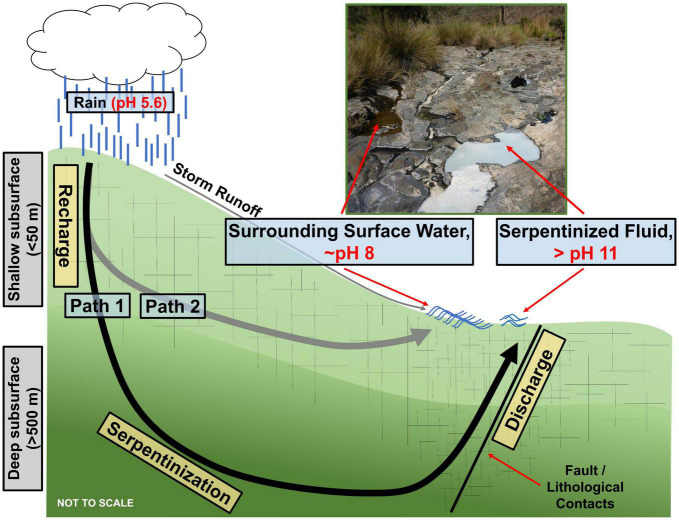
Conceptual model of serpentinization in the Samail Ophiolite of Oman adapted from Howells et al. (2022). Path 1 represents the generation of hyperalkaline (>pH 11) serpentinized fluid through infiltration of meteoric water to the deep subsurface where active serpentinization occurs. The reacted fluid is shown being expressed at the surface along faults and fissures. Path 2 represents meteoric water infiltrating the shallow subsurface and reacting to form ∼ pH 8 fluid. The image is of a serpentinized fluid study site (140111F) flowing next to and mixing with a surrounding surface water study site (140111G).

In this study the diversity of protists in serpentinization-hosted ecosystems in the Samail Ophiolite of Oman is explored through 18S rRNA gene amplicon sequencing. Additionally, the influence that geochemical and biological factors distinct to serpentinization in the Samail Ophiolite have on protist diversity are investigated. While there are many serpentinization-hosted ecosystems across the globe, the Samail Ophiolite in Oman offers serpentinized fluids that are easily accessible and relatively pristine due to the arid climate. Additionally, serpentinized fluid in the Samail Ophiolite can mix with surrounding surface water, which results in steep geochemical gradients that likely impact and shape the protist communities in the underlying sediments. Figure 1 contains a schema adapted from Howells et al. (2022) of the geological and geochemical context of serpentinization in Oman. Path 1 illustrates that water from the surface can infiltrate the deep subsurface (>500 meters) and react with ultramafic rock. The resulting fluids are hyperalkaline (pH > 11), reduced and depleted in dissolved inorganic carbon (DIC) and silica. Path 2 shows that surface fluids can also seep into the shallower subsurface (<50 meters) and react with rocks that have undergone a greater extent of serpentinization due to their proximity to the surface. In comparison to fluids that reacted with rocks in the deep subsurface, path 2 fluids are only slightly alkaline (∼ pH 8) and have higher concentrations of DIC and silica. As Figure 1 illustrates, reacted fluids can make their way to the surface following fault lines and fissures. Once expressed at the surface, path 1 and path 2 fluids can mix, creating gradients in pH, silica, DIC, O_2_ and H_2_. It was demonstrated that the distribution of hydrogenotrophic prokaryote communities corresponds strongly to variations in these geochemical parameters (Howells et al., 2022). The purpose of this study is to determine if protist diversity also reflects these geochemical variations.

The pH gradient (∼8 to >11) established by mixing between serpentinized fluid and surrounding surface water in Oman provides an opportunity to assess the influence alkaline pH has on microbial eukaryote diversity. In alkaline environments life can be proton limited, which has consequences for organisms acquiring energy through oxidative phosphorylation (Hicks et al., 2010). Therefore, alkaline environments can select for life that has adaptations for coping with proton limitation. While specific adaptations to alkaline pH have not been characterized in protists, ciliates are known to live in alkaline environments such as hypersaline soda lakes (Hu, 2014). In fact, the ciliate species *Frontonia anatolica* was discovered in the largest alkaline soda lake, Lake Van in Turkey (Yildiz and Şenler, 2013). In a study on ciliate populations living in two Kenyan soda lakes, Lake Bogoria (∼pH 10) and Lake Nakuru (∼pH 10), the species *Cyclidium glaucoma* was determined to be the most abundant ciliate and species of the genera *Frontonia*, *Condylostoma*, and *Holophyra* make up most of the biovolume (Ong’ondo et al., 2013). It should be noted that in addition to being alkaline, the Kenyan soda lakes are also hypersaline with Na^+^ being the dominant cation, and carbonate rich, which makes them distinct from serpentinized fluids. Overall, the ability of ciliates to inhabit soda lakes suggests that microbial eukaryotes may live in diverse alkaline environments.

There is some evidence that protists can live in serpentinization-hosted ecosystems, specifically the deep-sea hydrothermal field, Lost City, located near the Mid-Atlantic Ridge. López-García et al. (2007) conducted 18S rRNA gene sequencing on DNA extracts from carbonate samples collected from vent chimney walls and from vent fluids. Ciliates were found to be the dominant protists in both sample types. Amaral-Zettler (2013) conducted a survey of microbial eukaryote community composition using 18S rRNA gene sequencing across systems ranging from a pH 2 to pH 11 and included pooled DNA extracts from Lost City chimney wall and biofilm samples. The dominant protist phylotype in the Lost City samples is of the ciliate genus, *Hypotrichia*. The detection of ciliates at Lost City implies that ciliates may be well suited for hyperalkaline serpentinizing systems on the ocean floor. Serpentinized fluids in Oman provide an opportunity to assess if the same is true in continental serpentinizing systems. Additionally, the pH of serpentinized fluids in Oman varies greatly within the alkaline range, which provides an opportunity to assess the influence pH may have on protist diversity.

In addition to pH, other environmental factors that change along the Samail Ophiolite mixing gradient were explored by focusing on the physiological requirements of protists that are governed by their lifestyles. Protists have diverse lifestyles, with lineages capable of carrying out oxygenic photosynthesis, aerobic heterotrophy, and fermentation under microaerophilic and anaerobic conditions. The physiological constraints and resource demands for sustaining these lifestyles may have consequences for the distribution of protists along chemical gradients in Oman. Factors that influence protist distribution patterns may in turn influence protist community assembly and diversity at our sampling sites. For example, photosynthetic protists, like green algae and diatoms, have high demands for CO_2_ (Sültemeyer et al., 1988; Hu and Gao, 2008; Yamano and Fukuzawa, 2009). CO_2_ can be scarce in hyperalkaline serpentinized fluids and as a result may limit phototroph growth. Predatory heterotrophic protists are known to be selective in their prey (Straile, 1997; Seyler et al., 2019), feeding on smaller protists as well as bacterial and archaea (Pernthaler, 2005). In the sediments of serpentinized fluids of the Samail Ophiolite, bacterial and archaeal distributions are influenced by geochemical variations (Howells et al., 2022), which may have consequences for heterotrophic protists diversity. Some protists can carry out fermentation facultatively and some are strictly fermentative (Bernard and Fenchel, 1996). Either way, fermentation is an anaerobic process, and protists that depend on this metabolism are constrained to low oxygen environments (Fenchel and Finlay, 1990). We have demonstrated that factors including CO_2_, O_2_, and potential bacterial and archaeal foods sources change along the Samail Ophiolite mixing gradient (Howells et al., 2022). Guided by previous studies on the physiology and physiochemistry of phototrophic, heterotrophic, and anaerobic/fermentative protists, we assess whether the variations in the geochemical and biological factors observed in Howells et al. (2022) influence protist community composition and diversity. As there is increasing interest in the application of the natural process of serpentinization to carbon sequestration (Kelemen et al., 2011), understanding the diversity of microbial eukaryotes that both consume and produce CO_2_ in serpentinizing systems will be key to determining the overall efficacy of serpentinization for carbon sequestration.

## 2. Materials and methods

### 2.1. Study sites

Geochemical and biological samples were collected at 19 fluidic sites located at 6 different locations within the Samail Ophiolite of Oman, as summarized in Table 1. Sites are described as serpentinized fluid [Type 2 fluids in Leong et al. (2021)], surrounding surface water [Type 1 fluids in Leong et al. (2021)] and mixing in Table 1. A geologic map of the Samail Ophiolite of Oman that shows the locations is in Supplementary Figure 1. This study includes a subset of sites described in Howells et al. (2022). Protists were not detected through 18S rRNA gene amplicon sequencing at some of the sites described in Howells et al. (2022).

**TABLE 1:** Overview of sites in this study.

Sample identification		Field measurements		Mixing indicator		Site description
**Code**	**Location**	**Temp.**	**pH**	**Si**	**Percent**	
		**C°**		**μm**	**Serpentinized fluid**	
140115Y	Al Bana	24.5	11.6	2.16^a^	99.7	Serpentinized fluid
140114V	Falaij North	24.4	11.4	2.75 ^a^	99.5	Serpentinized fluid
140114T	Falaij North	27.2	11.4	1.84^a^	99.8	Serpentinized fluid
140113O	Falaij South	28.4	11.4	3.68^a^	99.2	Serpentinized fluid
140111G	Qafifah	22.6	8.9	276.35^a^	8.7	Visible mixing at surface
140111H	Qafifah	20.2	10.2	145^a^	52.3	Visible mixing at surface
140111I	Qafifah	18.8	10.9	100.25^a^	67.2	Visible mixing at surface
140111F	Qafifah	23.8	11.6	10.52^a^	96.9	Visible mixing at surface
140116B	Shumayt	26.5	7.9	302.59^a^	0.0	Surface water
140116D	Shumayt	27.1	9.1	260.49^a^	14.0	Visible mixing at surface
140117I	Shumayt	32.3	11.3	1.96^a^	99.8	Serpentinized fluid
140117G	Shumayt	30.5	11.4	1.50^a^	99.9	Serpentinized fluid
140117F	Shumayt	26.2	11.5	2.65^a^	99.5	Serpentinized fluid
140117H	Shumayt	29.6	11.5	1.66^a^	99.9	Serpentinized fluid
140110B	Dima	23.5	8.4	183.39^a^	39.6	Surface water
140112L	Dima	21.3	9.8	111.13^a^	63.5	Visible mixing at surface
140110D	Dima	21.8	10.4	91.38^a^	70.1	Visible mixing at surface
140112M	Dima	28.2	11.4	5.6^a^	98.6	Serpentinized fluid
140110C	Dima	27	11.4	4.26^a^	99.0	Serpentinized fluid
Geochemical model		40*	11.8*	1.39*	100	Chrysotile-brucite-diopside-calcite equilibria, 10 m*m* NaCl

This table is modified from Howells et al. (2022). The extent of mixing between surrounding surface water and serpentinized fluid is indicated by the calculated percent serpentinized fluid column. The site description notes locations where mixing between surrounding surface water and serpentinized fluid were visually observed. The locations of each of the sites can be found in the map in Supplementary Figure 1. The Geochemical model, reported in Leong et al. (2021), is of pristine serpentinized fluid derived from chrysotile-brucite-diopside-calcite equilibria and is used as our serpentinized fluid end member for calculating percent serpentinized fluid.

^a^Si values and methods for analyzing Si are reported in Leong et al. (2021).

*Values from model described in Leong et al. (2021).

### 2.2. Chemical analysis of fluids

Methods described by Leong et al. (2021) were used to measure temperature, pH, conductivity, dissolved oxygen (O_2_), and total dissolved Si and sulfide in the field. Samples were collected for lab analysis of major anions (F^–^, Cl^–^, SO_4_^2–^, NO_3_^–^), major cations (Li^+^, Na^+^, K^+^, Ca^2+^, Mg^2+^, NH_4_^+^), dissolved inorganic carbon (DIC), trace elements and dissolved gases. The concentrations of major ions, DIC, Si and H_2_ can be found in Table 2. Howells et al. (2022) describes the methods for dissolved H_2_ gas sample collection and analysis. Combined, the chemical data was used to calculate activities and solute speciation, as well as chemical affinities for mineral dissolution reactions with the geochemical modeling code EQ3 (Wolery and Jarek, 2003; Wolery and Jove-Colon, 2004), as described by Leong et al. (2021).

**TABLE 2:** Geochemical parameters of this study.

Site	Conductivity	pH	Si	Ca^+2^	H_2_	O_2_	DIC
**ID**	**μS/cm**		**μmolality**	**μmolality**	**μmolality**	**μmolality**	**ppm C**
140115Y	2778	11.6	2.16	1791.98	20.79	140.63	1.30
140114V	1803	11.4	2.75	1714.82	0.17	190.63	2.77
140114T	2061	11.4	1.84	1914.58	27.85	16.31	0.50
140113O	2329	11.4	3.68	2085.33	39.20	59.38	0.69
140111G	586	8.9	276.32	358.64	0.24	221.88	49.27
140111H	597	10.2	144.99	214.46	1.10	278.14	21.56
140111I	683	10.9	100.24	367.83	2.07	231.26	10.15
140111F	1470	11.6	10.52	1687.89	263.16	8.59	0.49
140116B	760	7.9	302.56	562.09	0.06	125.01	61.52
140116D	777	9.1	260.47	715.42	12.49	215.63	47.75
140117I	2000	11.3	1.96	1859.03	227.35	12.50	0.56
140117G	2015	11.4	1.50	1899.89	2.75	9.88	0.22
140117F	1868	11.5	2.65	1848.04	264.61	25.00	0.35
140117H	2067	11.5	1.66	2078.97	225.40	21.88	0.35
140110B	568	8.4	183.37	485.01	0.01*	243.76	43.60
140112L	659	9.8	111.12	312.81	0.01*	253.13	22.57
140110D	778	10.4	91.38	475.47	0.06	203.13	9.75
140112M	2058	11.4	5.60	1978.86	30.69	15.78	0.62
140110C	2011	11.4	4.26	1958.47	6.42	17.91	0.64

Geochemical parameters directly influenced by the process of serpentinization are pH, Si, Ca^+2^, H_2_, and DIC. pH, Si, O_2_, and DIC are investigated in this study as factors that may influence protist diversity. Ca^+2^ is relevant to the chemical speciation of DIC. H_2_ is relevant to potential interactions between anaerobic protists and hydrogenotrophic prokaryotes. Conductivity is provided for additional context.

*Denotes site where standard error on H_2_ measurement is greater than 5%. DIC, dissolved inorganic carbon.

### 2.3. DNA sample collection and extraction

At each sampling site, sediments underlying the site fluid were collected using a stainless-steel spatula that was washed with ethanol and flame sterilized. Sediment composition ranged from small rocky granules to silt. Sediment was sampled to up to approximately one centimeter in depth. 50 mL sterile specimen cups were filled to approximately 40 mL with sediment, which was then homogenized by stirring with the spatula, followed by shaking the sealed specimen cup. Homogenized sediment was then aliquoted into 2 mL cryo vials, and frozen on liquid nitrogen in the field. Frozen sediment samples were shipped to the lab in a dry shipper (still frozen) and placed in a −80°C freezer for storage.

The protocol used for DNA extraction, modified from methods described in Huber et al. (2002), is described in Howells et al. (2022) and included both physical and chemical lysis. Briefly, excess fluid was removed from sediment samples by spinning the cryo vials at 8000 rpm for 20 min at 10°C and removing the supernatant using filtered pipette tips. After transferring and weighing sediment samples in DNAse/RNAase-free 15 mL falcon tubes, lysis buffer was added at 1 mL per gram of sediment, and the samples were subjected to three freeze-thaw cycles. After physical lysis, samples went through chemical lysis which involved a lysozyme treatment (18 μL of 50 mg/mL stock per mL of extraction volume) followed by a proteinase K plus sodium dodecyl sulfate (SDS) treatment (22.5 μL of 20 μg/mL proteinase K stock and 45 μL of 20% SDS stock per mL of extraction volume). DNA was purified using the phenol/chloroform/isoamyl alcohol (24:24:1) method, precipitated with ethanol, dried overnight and washed according to the washing step from MP Biomedicals FastDNA Spin Kit for Soil. A final clean-up step was conducted using MO BIO PowerClean DNA Clean-Up kit.

### 2.4. Sequencing library preparation and sequencing

Amplicons of 16S and 18S rRNA genes were sequenced using the Illumina MiSeq v3 2 × 300 platform at the Arizona State University (ASU) Biodesign Institute core facilities. Sequencing library preparation followed a two-step PCR protocol with dual indexing (Kozich et al., 2013). The first-step PCR for the 16S rRNA gene used the forward general archaeal and bacterial primer 341F 5′-CCTACGGGNBGCASCAG-3′ (Takahashi et al., 2014), and the reverse general bacterial and archaeal primer 806R 5′-GGACTACNVGGGTWTCTAAT-3′ from the Earth Microbiome Project (Caporaso et al., 2011; Apprill et al., 2015; Parada et al., 2016). During the first-step PCR for the 18S rRNA gene the general eukaryote primer set with forward primer TAREuk454FWD1 5′-CCAGCASCYGCGGTAATTCC-3′ and reverse primer TAREukREV3 5′-ACTTTCGTTCTTGATYRA-3′ was used (Stoeck et al., 2010). The gene-specific primers included the addition of an overhang that allowed for the addition of barcodes and Illumina adapters in the second PCR (Hamady and Knight, 2009).

In the first PCR the template DNA was taken from the same sediment DNA extract for both the 16S rRNA and 18S rRNA gene amplifications. The first PCR thermocycler conditions for the 16S rRNA gene amplification were one denaturation cycle at 94°C for 3 min, 30 cycles of denaturation at 94°C for 30 s, annealing at 48°C for 30 s, and elongation at 72°C for 50 s. A final elongation cycle followed at 72°C for 10 min. The first PCR thermocycler conditions for the 18S rRNA gene amplification were the same for 16S, except for primer annealing at 47°C. The thermocycler conditions for the second PCR were a denaturation cycle at 95°C for 3 min, 30 cycles of denaturation at 95°C for 30 s, annealing at 50°C for 30 s, and elongation at 72°C for 50 s. A final elongation cycle followed at 72°C for 10 min. For each amplification PCR reagent mixtures included 15 μL EconoTaq 2x Master Mix, 2.5 μL of each primer (forward and reverse) for a final concentration of 2 μM, 2.5 μL template DNA, and 7.5 μL of sterile, nuclease free water for a final reaction volume of 30 μL. The PCR products were purified at the DNA Shared Resource Facility at ASU using Ampure magnetic beads in combination with a Beckman Biomek NXp robot. Purified products were quantified using Invitrogen Qubit broad range dsDNA fluorescent dye and a Biotek HT1 Plate Reader. After quantification, 25 ng of each amplicon sample, now having a unique barcode and Illumina adapters, were pooled. The pooled sample was then submitted for sequencing.

### 2.5. Sequencing data analysis

Paired-end sequencing data were demultiplexed at the ASU Genomics Facility and returned with quality analysis in the Casava 1.8 format as “.fastqc.gz” (FASTQC) files. Sequencing data from this project can be found in the NCBI Sequence Read Archive under the BioProject ID, PRJNA919024. Bioinformatic analysis of the FASTQC files was done using the QIIME2-2019.7 (RRID:SCR_021258) program wrapper (Bolyen et al., 2019). To generate amplicon sequence variants (ASVs), paired-end FASTQC files were quality filtered, trimmed, Illumina error corrected, merged and chimera checked using the program DADA2 (Callahan et al., 2016).

Taxonomic assignment of ASVs was done using a naïve Bayes trained classifier generated from the SILVA 132 99% OTU 16S and 18S databases (Quast et al., 2013) and the sklearn method in the QIIME 2 plugin, q2-feature-classifier (Pedregosa, 2011; Bokulich et al., 2018). After taxonomic assignment, 16S ASVs that occur only once were filtered out and 18S ASVs that occur less than 10 times across all sites were filtered out to be conservative in defining detectable phylotypes. 16S ASVs classified only to the domain level and those classified as species associated with the human microbiome were filtered out. 18S ASVs classified only to the domain level were filtered out. Because this study is focused on protist diversity, 18S ASVs classified as Opisthokonta and Magnoliophyta were filtered out. After filtering, sequence reads were rarefied for even sampling across sites; 16S reads were rarefied to 9,200 per site, and 18S reads were rarefied to 1,900 per site. Alpha rarefaction curves were made to ensure sufficient sampling of 16S and 18S diversity, as shown in Supplementary Figure 2. The rarefied reads were used to build a final frequency table of ASVs across all sites. Faith’s phylogenetic diversity analysis was carried out on both 16S and 18S rarefied ASVs using the q2-diversity plugin (Faith, 1992), after aligning ASVs using MAFT within q2-align (Katoh et al., 2002). A phylogeny of the aligned ASVs was constructed with FastTree2 within q2-phylogeny (Price et al., 2010). The rarefied frequency table and phylogenetic diversity analysis were used in the following statistical analyses.

### 2.6. Statistical analyses and graphing

Non-metric multidimensional scaling (NMDS) ordination, Mantel correlation analysis, and analysis of similarity (ANOSIM) were carried out using the vegan package (version 2.5-6, RRID:SCR_011950) within R version 3.6.0 (R Core Team, 2019). A dendrogram heatmap was made using the pheatmap package in R. Additional ordinations and similarity percentage (SIMPER) analysis were carried out using the program Past 4.02 (Hammer et al., 2001). Scatter plots, Pearson correlation analyses, and Mann–Whitney U-tests were carried out using OriginPro, Version 2019b (RRID:SCR_014212) made by OriginLab Corporation, Northampton, MA, USA. Rarefied ASV frequencies were converted to relative abundances per site to assess variations in composition of 16S and 18S rRNA gene ASVs. Relative abundances were square-root transformed, and Bray-Curtis dissimilarity matrices were generated using the vegan package. To assess drivers of 18S and 16S compositional changes, Mantel correlation analyses with 999,999 permutations were done using the Bray-Curtis dissimilarity matrices and Manhattan dissimilarity of log transformed geochemical parameters. NMDS ordinations of the Bray-Curtis dissimilarity matrices were carried out with 1,000 permutations and two dimensions (*k* = 2). For both the 18S and 16S ASV NMDS plots, the stress was less than 0.1. The NMDS stress plot for 18S ASVs is shown in Supplementary Figure 3A. 95% concentration ellipses were drawn on principle coordinates analysis (PCO) and NMDS ordination of 18S ASVs in Past 4.02 (RRID:SCR_019129) to assess ASV-driven formation of site clusters as shown in Supplementary Figures 3B, C. Tests of correlations between geochemically defined groups and the observed clusters from the 95% concentration ellipse analyses were carried out with ANOSIM using 9,999 permutations. SIMPER analysis was used to evaluate the contribution of each 18S ASV to the Bray-Curtis dissimilarity between the 95% concentration ellipse clusters and sites grouped based on their geochemical composition. Mann–Whitney U-tests were conducted to test if differences in relative abundances of microbial groups correspond to differences in geochemical composition among sites. Correlations between geochemical parameters and the observed number of ASVs of microbial groups (richness) were evaluated using scatter plots and Pearson correlation analysis in Origin 2019b. Mann–Whitney U-tests were also conducted to assess the correspondence between variations in richness and the geochemically defined site groups.

## 3. Results

### 3.1. Geochemical composition of site fluids

Figure 1 illustrates that in Oman there are two distinct fluid compositions, one being what we call surrounding surface water, which is ∼ pH 8, the other being serpentinized fluid, pH > 11. The sediments we sampled for characterization of protist diversity have overlaying fluids that include the two distinct fluid types as well as mixtures of the two. The fluid type at each of our sampling sites is noted in Table 1 together with measurements of silicon (Si), which can be used as an indicator of the extent of serpentinization (Leong and Shock, 2020; Leong et al., 2021). Additionally, because the concentration of Si in serpentinized fluid expressed at the surface is nearly 3 orders of magnitude lower than the concentration in surrounding surface water, Si can be used as an indicator of the extent of mixing between the two (Leong et al., 2021). Therefore, we followed Howells et al. (2022) and used a linear mixing model with Si concentrations to calculate the percent contribution of serpentinized fluid to each sampling site. One end member of the model is a surface water site from this study with the highest concentration of Si (site 140116B) and the other is a geochemical model of a pristine serpentinized fluid reported in Leong et al. (2021). For results from this calculation, see Table 1. Percent serpentinized fluid is referenced throughout the text below to help define sampling along the mixing gradient in the Samail Ophiolite of Oman.

Measurements of conductivity, the concentrations of the calcium ion (Ca^+2^), dissolved H_2_, dissolved O_2_ and dissolved inorganic carbon (DIC) are listed in Table 2, together with the pH and Si values reported in Table 1. Scatter plots of pH, DIC, O_2_ and H_2_ plotted as function so Si (indicator of mixing) can be found in Supplementary Figure 4. We include measurements of Ca^+2^ as chemical modeling shows that aqueous CaCO_3_ and CO_3_^–2^ are the most abundant inorganic carbon species in serpentinized fluids (see Supplementary Figure 5 and Supplementary Table 1).

Howells et al. (2022) observed a distinct compositional change in sediment archaeal and bacterial (prokaryote) diversity and overlying fluid chemistry that corresponds to whether fluids with greater than or less than 70% serpentinized fluid. Therefore, we conducted non-parametric Mann–Whitney U-tests to assess whether pH, O_2_ and DIC at sites above and below 70% serpentinized fluid composition are significantly different. Those results are summarized in Table 3.

**TABLE 3:** Summary of Mann–Whitney U-tests conducted on the relative abundance and richness of protist and prokaryote microbial groups and fluid geochemistry.

	Factor	Test description	Min	Median	Max	Min	Median	Max	*U*-value	*Z*-value	Exact *p*-value
			**Group 1 (G1), *N* = 12** **>70% serpentinized fluid**			**Group 2 (G2), *N* = 7** **<70% serpentinized fluid**					
Geochemistry	pH	G1 > G2	10.4	11.4	11.6	7.9	9.1	10.9	83	3.4	4.0E−05
	Si (μmolality)	G1 < G2	1.5	2.7	91.4	100.2	183.4	302.6	0	−3.5	2.0E−05
	Ca^+2^ (μmolality)	G1 > G2	475.5	1879.5	2085.3	214.46	367.8	715.4	81	3.3	1.4E−04
	H_2_ (μmolality)	G1 > G2	0.06	29.3	264.6	0.01	0.2	12.5	73	2.6	3.6E−03
	*DIC (μmolality)	G1 < G2	18.6	49.3	811.7	845.36	3630.5	5122.6	0	−3.5	2.0E−05
	O_2_ (μmolality)	G1 < G2	8.6	19.9	203.1	125.01	231.3	278.1	3	−3.3	1.4E−04
Relative abundance	Phototrophic protists	G1 < > G2	0	6.6	62.8	31.6	65.1	75.1	9	−2.8	3.7E−03
	Heterotrophic protists	G1 < > G2	3.9	66.6	96.4	18.5	33.4	58.1	63	1.7	8.3E−02
	Anaerobic protists	G1 < > G2	0.5	11.9	87.3	0	0.2	3.5	81	3.3	2.8E−04
	*Chlorophyceae*	G1 < G2	0	0.1	5.4	0.2	1.9	13.4	15	−2.3	9.7E−03
	*Cyclidium*	G1 > G2	0.4	10.7	87.3	0	0	3.5	82	3.4	7.9E−05
	*Ischnamoeba montana*	G1 > G2	0.1	12.3	67.3	0	0	0.6	81	3.3	1.4E−04
	*Diatomea*	G1 < G2	0	0.1	10.4	6.2	29.4	54.6	1.5	−3.4	6.0E−05
Richness	All Protists	G1 < G2	9	18	111	103	154	179	1	−3.4	4.0E−05
	Phototrophic protists	G1 < G2	0	4	41	39	58	64	1	−3.4	4.0E−05
	Heterotrophic protists	G1 < G2	3	11.5	62	52	84	101	3	−3.3	1.4E−04
	Anaerobic protists	G1 < > G2	1	2	4	0	1	7	47.5	0.4	6.8E−01
	All prokaryotes	G1 < G2	67	97	200	67	504	831	11.5	−2.5	3.8E−03

Groupings include sites with > and <70% serpentinized fluid composition. Relative abundance refers to the relative abundance of the 16S or 18S rRNA gene ASVs. Richness refers to the observed number of ASVs. The *U*-value is the test statistic for the Mann–Whitney U test. The *Z*-value is the approximate normal test statistic derived from the *U*-value. *U* and *Z*-values were calculated using OriginPro Version 2019b.

*DIC, dissolved inorganic carbon. For an overview of the modeled inorganic carbon activities, see Supplementary Table 1.

### 3.2. Protist taxonomy

**FIGURE 2:**
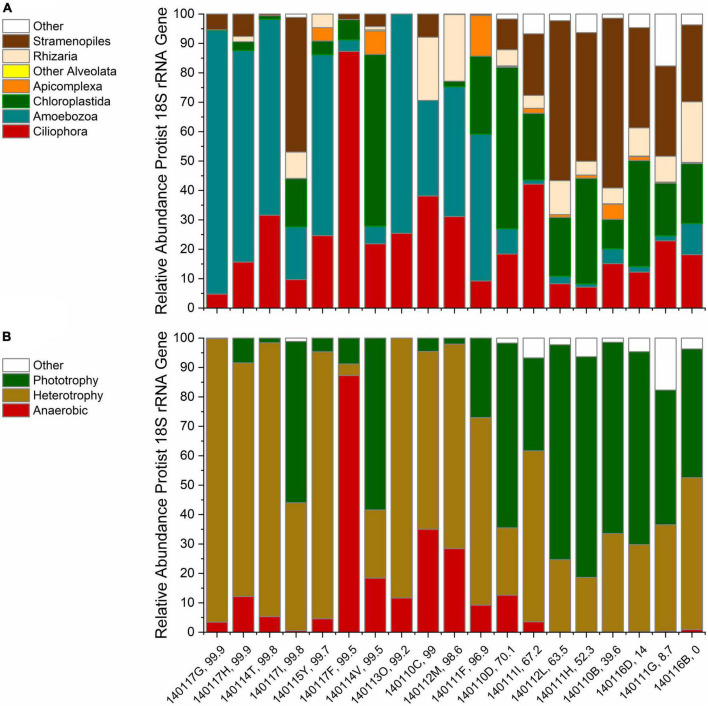
Rarefied protist ASV relative abundances. Each bar is labeled with the sampling site identification and the percent serpentinized fluid composition of the sampling site water. The bars are ordered by increasing percent serpentinized fluid from right to left. **(A)** Relative abundances arranged in order of decreasing contribution of serpentinized fluid to water composition at each site using taxonomic classification by major protist groups described in Adl et al. (2019) and the SILVA 32 database. Other Alveolata includes ASVs classified as Colpodellida and Perkinsidae. **(B)** Bar chart of ASV relative abundances grouped by lifestyles of the inferred taxa arranged as in panel **(A)**.

We classified the protist 18S rRNA gene ASVs taxonomically to explore variations in distribution and diversity of protist phylotypes grouped by taxa and lifestyle. An overview of the relative abundances of phylotypes grouped by the taxonomy reviewed in Adl et al. (2019) is given in Figure 2A. The bar chart is ordered by decreasing contribution of serpentinized fluid to each sampling site fluid composition. Stramenopiles and Chloroplastida phylotypes are the most abundant phylotypes at sites that have <70% serpentinized fluid. At sites with >70% serpentinized fluid, many communities are dominated by Amoebozoa phylotypes. Ciliophora phylotypes are present throughout the gradient and make up nearly 90% of the community at a site with 99.5% serpentinized fluid composition (site 140117F). Rhizaria and Apicomplexa are scattered along the gradient. At three sites with >70% serpentinized fluid (sites 140117I, 140114V, and 140110D) the protist communities have similarities to those from sites with <70% serpentinized fluid, having large relative abundances of Chloroplastida and in one case Stramenopile phylotypes. Having filtered out Opisthokonta for the diversity analyses we did exclude some protist ASVs taxonomically classified as Chanoflagellida. In the pre-filtered data set, these ASVs occur only at sites with <70% serpentinized fluid.

Protist phylotypes were grouped by their respective lifestyles, which was determined through descriptions of the taxonomic groups in the literature (Adl et al., 2019). For this study we focus on phototrophic, heterotrophic and anaerobic lifestyle types. Figure 2B shows the relative abundances of protist 18S rRNA gene ASVs grouped by lifestyle. Note that phototrophs are abundant at most sites with <70% serpentinized fluid. Anaerobic protists are most abundant at sites with >70% serpentinized fluid. Heterotrophs are distributed throughout the mixing gradient and show higher relative abundances in locations with elevated contributions of serpentinized fluid. The differences in distribution are reflected in Table 3, which reviews Mann–Whitney U-tests conducted to evaluate whether there are significant differences in the distribution between sites with greater than 70% and less than 70% serpentinized fluid.

### 3.3. Alpha diversity of protists

In this study we measured alpha diversity (richness) in two ways. One way is the count of the number of distinct 18S rRNA gene ASVs that occur at each site which we refer to as the observed number of ASVs. Another assessment of alpha diversity employed in this study is phylogenetic diversity calculated using the Faith method (Faith, 1992). This approach normalizes the richness of 18S rRNA gene sequences by the branch lengths of a phylogenetic tree constructed from the ASVs (see the section “2. Materials and methods”). While we report phylogenetic diversity, it is not a major focus of this study. Richness and phylogenetic diversity values at each site are reported in Table 4, which includes the initial count and counts of sequences at each stage of processing and filtering during sequence analysis. As noted in the section “2. Materials and methods” Supplementary Figure 2 includes the rarefaction curves of filtered sequences at each site used to ensure sufficient sampling depth. Richness and phylogenetic diversity of 16S rRNA genes (prokaryotes) at each site are also reported in Table 4. The rarefaction curves for both the 18S and 16S rRNA gene reads indicate that the sites with the highest observed number of ASVs, 140111G, may not have sufficient sampling depth and therefore likely has more diversity than is represented in this study.

**TABLE 4:** Read depth, observed number of ASVs and phylogenetic diversity.

Site ID	Raw sequence frequency	DADA2 pipeline frequency	Non-chimeric frequency	Contaminants filtered frequency	*Observed number of ASVs (richness)	Faith’s phylogenetic diversity
**18S rRNA gene amplicon**						
140117F	13850	6201	6201	5155	9	2.5
140117I	47452	38112	38102	13793	66	12.6
140116D	46408	37535	37316	12234	103	16.4
140117G	30809	24086	23935	7913	24	4.8
140116B	31831	24303	24139	12588	162	20.8
140113O	38718	27165	27143	5902	14	2.8
140115Y	27152	20089	19992	5315	19	5.3
140114T	23568	15160	15151	5711	17	3.5
140112L	36633	28460	28350	14448	130	18.3
140114V	45712	35596	34858	16928	44	5.7
140112M	29895	23347	22939	12288	23	5.2
140111I	152131	122182	113625	74206	154	18.6
140111H	43126	34155	33080	15734	126	18.1
140111G	86303	68413	68019	45861	179	24.4
140111F	32311	25667	24915	1932	14	4.1
140110C	43351	31847	25422	2198	11	3.4
140110D	18013	14036	13994	9808	111	15.7
140110B	48617	37795	36160	15770	156	21.8
140117H	26691	21440	21440	2262	16	4.4
**16S rRNA gene amplicon**						
140117F	44593	36561	35122	35114	91	16.1
140117I	31398	25253	24518	24239	200	30.1
140116D	52559	37017	34962	34466	451	47.9
140117G	28442	22341	20661	20659	116	18.7
140116B	64415	39181	38115	35828	831	78.0
140113O	26672	19437	17936	17844	67	15.1
140115Y	20065	15587	14471	14471	86	17.6
140114T	56169	43834	36543	36510	137	21.2
140112L	43196	24496	23400	22495	504	48.0
140114V	33551	25072	21807	21781	85	14.2
140112M	29764	22764	20979	20734	111	20.2
140111I	32875	17727	16836	16717	429	44.3
140111H	19067	9850	9662	9255	402	46.7
140111G	45400	27797	27400	25992	760	73.2
140111F	20835	16042	13522	13475	81	14.7
140110C	67207	53346	52903	52687	163	27.0
140110D	21339	16732	16163	16129	91	18.1
140110B	41080	24393	23506	20264	762	73.2
140117H	22711	17814	17058	17013	103	19.0

*Values for the observed number of ASVs are after rarefaction.

Using Pearson correlation analysis, protist richness was compared with geochemical and biological parameters that we hypothesize influence protist diversity in sediments of serpentinized fluid. Specifically, we evaluated correlations between overall observed number or protist ASVs and pH, Si (log transformed) and observed number of prokaryote ASVs, all of which are significant (Pearson *r*-value > 0.70 and ANOVA *p*-value > 0.005) as summarized in Table 5. We also evaluated correlations between 1) observed number of phototrophic protist ASVs and the concentration of DIC, and 2) observed number of heterotrophic protist ASVs with the concentration of O_2_ and the observed number of prokaryote ASVs. Phototrophic protist richness was found to correlate significantly with DIC. Heterotrophic protist richness was found to correlate significantly both with the concentration of O_2_ and to a greater extent, the observed number of prokaryote ASVs (see Table 5 for Pearson *r*-values and ANOVA *p*-values).

**TABLE 5:** Pearson correlations between geochemical and biological factors and observed number of protist (18S rRNA gene) ASVs.

Factors	Richness metric	Pearson *r*-value	ANOVA *p*-value
pH	Observed number protist ASVs	−0.86	2.5E−06
Si (log molality)	Observed number protist ASVs	0.92	4.0E−08
Si (log molality)	Phylogenetic diversity protist ASVs	0.91	5.5E−08
DIC (molality)	Observed number phototrophic protist ASVs	0.92	9.0E−09
O_2_ (molality)	Observed number heterotrophic protist ASVs	0.77	1.2E−04
Si (log molality)	Observed number prokaryotic ASVs	0.84	9.1E−06
Observed number prokaryote ASVs	Observed number heterotrophic protist ASVs	0.85	5.1E−06
Observed number prokaryote ASVs	Observed number protist ASVs	0.9	3.4E−07

Mann–Whitney U-tests were also carried out to determine if there is significant difference in protist richness between those sites with >70% serpentinized fluid and those with <70% serpentinized fluid. As summarized in Table 3, protist richness is significantly higher at sites with <70% serpentinized fluid than at sites with >70% serpentinized fluid. When examining protist richness grouped by lifestyle, the same holds true for phototrophic and heterotrophic protists. Anaerobic protist richness is overall much lower than phototrophic and heterotrophic richness and is not significantly different between sites with >70% serpentinized fluid and those with <70% serpentinized fluid.

### 3.4. Beta diversity of protists

We evaluated variations in community composition, or beta diversity, by conducting Bray-Curtis dissimilarity analysis of the relative abundances of protist 18S rRNA gene ASVs. The relative abundances were square-root transformed. We conducted Mantel correlation analysis between the protist dissimilarity matrix and (1) Manhattan dissimilarity of geochemical parameters and (2) Bray-Curtis dissimilarity of square-root transformed 16S rRNA gene relative abundances. The r-statistic and *p*-value for each Mantel correlation analysis are reported in Table 6. All Mantel correlations are significant with *p*-values < 0.005. The correlations with relatively lower r-statistics are between protists and pH (r-statistic = 0.62), phototrophic protists and dissolved inorganic carbon (DIC) (r-statistic = 0.42) and heterotrophic protists and dissolved O_2_ (r-statistic = 0.53). Highest Mantel correlations are between protists and the concentration of Si (r-statistic = 0.71), prokaryotes and the concentration of Si (r-statistic = 0.70), heterotrophic protists and prokaryotes (r-statistic = 0.71) and protists and prokaryotes (r-statistic = 0.73).

**TABLE 6:** Mantel correlations between dissimilarity of geochemical and biological factors and dissimilarity of protist 18S rRNA gene ASV relative abundances.

Factors	Microbial group	r-statistic	*p*-value
Manhattan, pH	Bray-Curtis, rarified protist ASV relative abundances (square-root transform)	0.62	2.0E−06
Manhattan, Si (log molality)	Bray-Curtis, rarified protist ASV relative abundances (square-root transform)	0.71	1.6E−05
Manhattan, DIC (log molality)	Bray-Curtis, rarified phototrophic protist ASV relative abundances (square-root transform)	0.42	5.3E−04
Manhattan, O_2_ (molality)	Bray-Curtis, rarified heterotrophic protist ASV relative abundances (square-root transform)	0.53	1.2E−04
Manhattan, Si (log molality)	Bray-Curtis, rarified prokaryote ASV relative abundances (square-root transform)	0.70	1.0E−06
Bray-Curtis, rarified prokaryote ASV relative abundances (square-root transform)	Bray-Curtis, rarified heterotrophic protist ASV relative abundances (square-root transform)	0.71	2.0E−06
Bray-Curtis, rarified prokaryote ASV relative abundances (square-root transform)	Bray-Curtis, rarified protist ASV relative abundances (square-root transform)	0.73	2.0E−06

As noted in the table, Manhattan dissimilarity was applied to geochemical factors. Bray-Curtis dissimilarity was applied to protist ASV relative abundances. Any transformations applied to the data are noted. DIC stands for dissolved inorganic carbon.

## 4. Discussion

### 4.1. Protist diversity as a function of pH

The variation in pH along the mixing gradient observed is in large part due to the process of serpentinization and subsequent mixing between serpentinized fluids and surrounding surface water (Leong et al., 2021). In previous studies on microbial diversity of serpentinized fluid of the Samail Ophiolite, archaeal and bacterial diversity was shown to decrease as pH increases [reviewed in Howells et al. (2022)]. We found the observed number of protist ASVs (or richness) also significantly decreases with pH (Pearson *r*-value = −0.86 and ANOVA *p*-value < 0.005). A significant correlation between Bray-Curtis dissimilarity of square-root transformed protist ASV relative abundances and Manhattan dissimilarity of pH values was also observed (Mantel r-statistic = 0.62, *p*-value < 0.005), which indicates that variation in protist community composition significantly corresponds to pH.

The significant anti-correlation of protist richness and significant correspondence of protist community composition variation with pH may be explained by proton limitation experienced by protists under alkaline conditions. To overcome proton limitation, bacteria have adaptations such as increased expression of Na^+^/H^+^ antiporters or specialized adaptations in ATP synthase (Hu and Gao, 2008). It follows that hyperalkaline pH requires specialized adaptations, some of which can be energetically costly to maintain. While there is not much known about protist capabilities for tolerating alkaline pH, it is possible only a few lineages are capable of surviving under such conditions. If true, this may explain the decrease in diversity, and differences in community composition, as pH increases.

### 4.2. Emerging ecotone

Howells et al. (2022) observed a distinct transition in archaeal and bacterial community composition in sediments in Oman at the point where the overlying water contains 70% serpentinized fluid. Specifically, sediments with overlying fluids that have >70% serpentinized fluid are populated by hydrogenotrophic bacteria and archaea that include aerobic hydrogen oxidizers, sulfate reducers and methanogens. The abundance of hydrogenotrophs drops off at sites with <70% serpentinized fluid and the overall bacterial and archaeal sediment community composition is significantly different among sites that fall above and below 70% serpentinized fluid. Additionally, bacterial and archaeal richness is significantly higher at sites with <70% serpentinized fluid. Howells et al. (2022) suggested the significant differences in pH, H_2_ concentration, and concentrations of electron acceptors used by hydrogenotrophs translates to the distinct transition in archaeal and bacterial diversity between sampling sites that have greater than 70% serpentinized fluid and sites with less than 70% serpentinized fluid.

**FIGURE 3:**
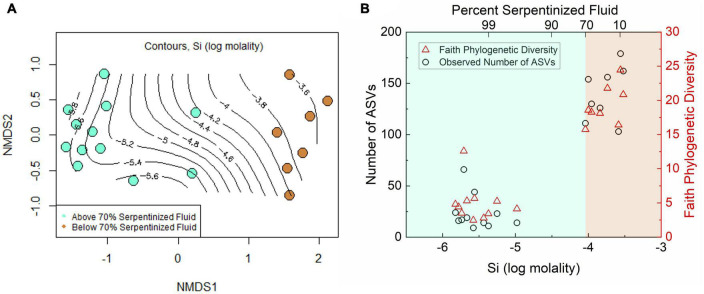
Diversity of protist ASVs along the Oman geochemical gradient. **(A)** Non-metric multidimensional scaling (NMDS) ordination of Bray-Curtis dissimilarity of rarefied, square-root transformed protist ASV relative abundances. Two dimensions were used. The stress is 0.094. The contours show Si concentration at each site and are drawn using a maximum likelihood method. Color indicates content of serpentinized fluid above and below 70%. Communities at sites above 70% serpentinized fluid composition are significantly different from those below (ANOSIM r-statistic = 0.93 and *p*-value < 0.001). **(B)** Richness, or the number of ASVs at each site, and Faith phylogenetic diversity of ASVs plotted against Si concentration. Communities at sites with water compositions <70% serpentinized fluid are significantly less rich than sites with >70% serpentinized fluid (Mann–Whitney U-test *p*-value < 0.05).

To investigate whether there is also a distinct transition in protist diversity we carried out NMDS ordination, as shown in Figure 3A, with Bray-Curtis dissimilarity of protist 18S rRNA gene ASV relative abundances (square-root transformed). The contours on the NMDS ordination show that variation along NMDS1 corresponds closely to the concentration of Si. A Mantel test between Manhattan dissimilarity of log Si concentration and Bray-Curtis dissimilarity of the relative abundances shows there is significant correlation between Si and community composition (Mantel r-statistic of 0.71, *p*-value < 0.005). As evident in Figure 3A, there are two distinct groups separated along NMDS1 that correspond to sites having less than (tan) or greater than (turquoise) 70% contribution from serpentinized fluid. Furthermore, ANOSIM shows that the transition in community composition at 70% serpentinized fluid is significant (ANOSIM r-statistic 0.93, *P* < 0.005), which also holds true for presence and absence of ASVs (Jaccard dissimilarity, ANOSIM r-statistic 0.93, *p*-value < 0.005). Supplementary Figures 3B, C show 95% concentration ellipses drawn on an NMDS plot and principal coordinate analysis (PCO) plot. The ellipses are estimates of where 95% of the ASVs are expected to fall based on their frequency and correspond to grouping of sites above and below 70% serpentinized fluid. In the NMDS plot, two sites with >70% serpentinized composition fall in the 95% ellipse that encompasses sites with <70% serpentinized fluid. This suggests that these two sites (140117I and 140110D), located centrally along NMDS1 in Figure 3A, are intermediate to the grouping above and below 70% serpentinized fluid composition.

Together with the ordination plots and the ANOSIM analysis, the ellipses suggest an overarching community type at sites with <70% serpentinized fluid and another type at sites with >70% serpentinized fluid, with some variability within the two groups. These results, along with observations made in Howells et al. (2022), demonstrate a sharp transition in community composition. The space of transition, or merging, between two distinct communities connected in geographical space is called an ecotone. In our study, the sampling sites are in different geographical locations of the Samail Ophiolite, therefore an ecotone in this setting may reflect a transition between two community types in a chemical space that results from serpentinization and fluid mixing. Protist taxa within the sites located centrally along NMDS1 in Figure 3A may reflect the environmental and biological dynamics of a chemical ecotone. We acknowledge more sampling at sites with around 70% serpentinized fluid would increase the confidence in the presence of a chemical ecotone in this study. Now that Si can be used to determine percent serpentinized fluid composition, analysis of Si in the field can be used to target potential ecotone sites in future biodiversity studies.

Like the variation in protist community composition, protist ASV richness also significantly varies with the concentration of Si as indicated by the Pearson correlation analysis with log Si, *r*-value = 0.92 and ANOVA *p*-value < 0.005. Richness is plotted as a function of log Si in Figure 3B and illustrates that the observed number of protist ASVs at sites with <70% serpentinized fluid are significantly higher than the observed number of ASVs at sites with >70% serpentinized fluid, which is supported by the Mann–Whitney U-test *p*-value < 0.05 (see Table 3).

**FIGURE 4:**
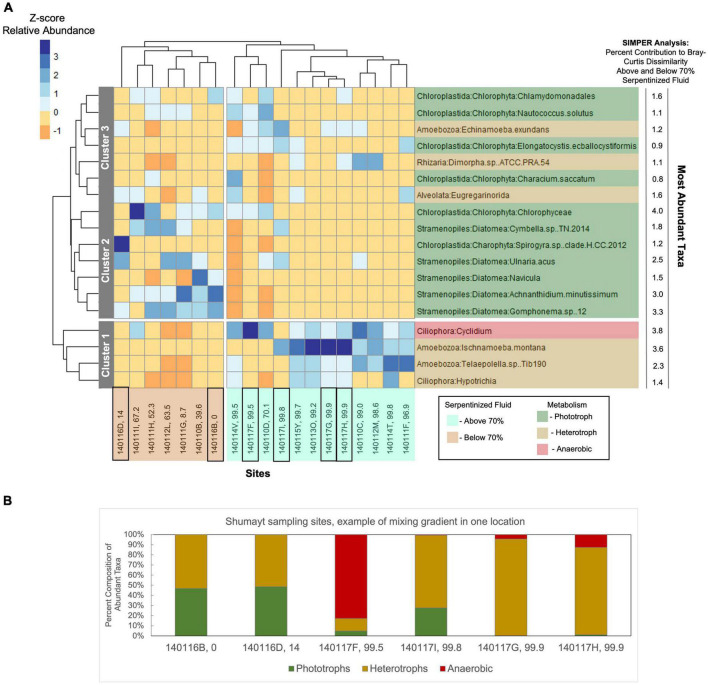
Heatmap of the most abundant taxa with cluster dendrogram analyses of both sites and taxa. **(A)** Taxa assigned at SILVA level 9 with >1% average relative abundance at all sites were included in the analysis. Relative abundances were square-root transformed. In the R package, pheatmap, Euclidean dissimilarity between sites was calculated based on the composition of the taxa and a complete cluster method was employed to construct the dendrogram shown at the top of the heatmap. For the dendrogram on the left of the heatmap, Euclidean dissimilarity between taxa was calculated based on the differences in distribution across sites and complete cluster analysis was used to construct the dendrogram. The coloration of the heatmap is scaled to z-scores of the relative abundances of taxa at each site. Two distinct clusters are revealed based on the composition of the most abundant taxa; one cluster composed of sites with water compositions that are >70% serpentinized fluid (cyan highlighted site labels) and the other composed of sites with <70% serpentinized fluid (brown highlighted site labels). Therefore, the compositions of the most abundant taxa align with overall community composition differences observed in the NMDS ordination of all protist 18S ASVs in Figure 3A. The taxa dendrogram on the left shows three distinct clusters. As shown by the heatmap coloration, cluster 1 is composed of taxa that are most abundant at sites with water containing >70% serpentinized fluid composition. Cluster 2 is composed of taxa that are most abundant at sites with <70% serpentinized fluid. Cluster 3 is composed of taxa that occur across the gradient regardless of water composition. Black squares around site IDs on the bottom of the dendrogram indicate Shumayt sampling sites. Shumayt is a location where serpentinized fluid and surrounding surface water co-occur and can mix, which may result in sites (e.g., 140117F and 140117I) with protist communities that reflect a chemical ecotone. **(B)** The composition of the most abundant taxa grouped by their lifestyles at Shumayt sampling sites.

We hypothesize that the distinct differences in protist communities between sites with >70% serpentinized fluid and sites with <70% serpentinized fluid can be explained by the physiological differences of protists based on their lifestyle, which dictate the properties of the niche they occupy. To test this hypothesis, we constructed a dendrogram heatmap shown in Figure 4A of protist phylotypes grouped by SILVA taxonomic level 9 whose average relative abundance across sites is greater than 1%. The relative abundances of these phylotypes at each site is given in Table 7 and the overall contribution of these taxa to each community is illustrated in Supplementary Figure 6. The site-based dendrogram shown at the top of the heatmap in Figure 4A is derived from a cluster analysis, using the complete method, of Euclidean dissimilarity among sites based on the compositions of the taxa (defined by their occurrence and relative abundance) and illustrates *how similar the sites are* based on the composition of the protist taxa. The taxa-based dendrogram on the left of the heatmap uses differences in abundances of taxa among sites and illustrates *how similar taxa are* based on their distribution. The colors in the heatmap reflect the composition of the most abundant taxa at each site. For example, in the heatmap, the dark blue square at the site with 99.5% serpentinized fluid (site 140117F) indicates that the most abundant taxon at this site is of the genus, *Cyclidium*. The site-based dendrogram at the top of Figure 4 shows two distinct clusters on either side of the 70% serpentinized fluid transition, consistent with the NMDS ordination in Figure 3A and the ANOSIM analysis discussed above. Therefore, the compositions of the most abundant taxa correspond to overall protist community dissimilarity above and below 70% serpentinized fluids.

**TABLE 7:** List of protist taxa grouped at SILVA 32 level nine taxonomy whose average relative abundance across sites is greater than 1% and their metabolism.

Taxa	140117G	140117H	140114T	140117I	140115Y	140117F	140114V	140113O	140110C	140112M	140111F	140110D	140111I	140112L	140111H	140110B	140116D	140111G	140116B
**Phototrophic, Chloroplastida, Charophyta**																			
*Spirogyra* sp. clade H.CC.2012	0	0	0	0	0	0	0	0	0	0	0	0	0	1	0	0	33	0	0
**Phototrophic, Chloroplastida, Chlorophyta**																			
*Characium saccatum*	0	0	0	0	0	0	23	0	0	0	0	0	0	0	1	1	0	0	0
Chlamydomonadales	0	2	0	1	0	0	7	0	0	0	0	6	1	0	4	0	0	0	4
*Nautococcus solutus*	0	1	0	0	0	2	7	0	0	0	0	15	0	2	2	0	0	2	0
*Chlorophyceae*	0	0	0	1	0	1	3	0	0	0	0	5	13	1	12	2	0	1	2
*Elongatocystis ecballocystiformis*	0	0	0	4	4	2	5	0	0	0	15	3	0	0	0	0	0	0	0
**Phototrophic, Stramenopiles, Diatomea**																			
*Achnanthidium minutissimum*	0	0	0	0	0	0	0	0	0	0	0	0	1	4	2	6	0	7	9
*Cymbella* sp. TN.2014	0	0	0	6	0	0	0	0	0	0	0	1	3	14	12	0	0	1	0
*Gomphonema* sp. 12	0	0	0	0	0	0	0	0	0	0	0	0	0	11	11	10	3	2	6
*Navicula*	0	0	0	0	0	0	0	0	0	0	0	1	0	1	0	24	0	0	1
*Ulnaria acus*	0	0	0	3	0	0	0	0	5	0	0	2	0	15	1	2	14	6	0
**Heterotrophic, Amoebozoa**																			
*Telaepolella* sp. Tib190	3	2	48	0	7	0	3	10	21	10	36	0	0	0	0	0	0	0	0
*Ischnamoeba montana*	67	64	15	10	45	1	0	62	8	33	6	1	0	1	0	0	0	0	0
*Echinamoeba exundans*	3	2	0	7	0	2	0	0	4	0	1	5	0	0	0	0	1	0	0
**Heterotrophic, other**																			
Alveolata, *Eugregarinorida*	0	0	0	0	3	0	8	0	0	0	10	0	1	0	1	4	1	0	0
Ciliophora, *Hypotrichia*	1	2	26	0	11	0	3	13	0	0	0	0	0	0	0	0	0	0	0
Rhizaria, *Dimorpha* sp. ATCC PRA.54	0	2	0	0	5	0	1	0	18	23	0	0	0	0	0	0	0	0	0
**Anaerobic (can be microaerophilic)**																			
Ciliophora, *Cyclidium*	3	11	5	0	5	87	18	10	35	28	9	13	4	0	0	0	0	0	0

Values in the table are the relative abundances of the taxa at each sampling site.

Insights into how the distributions of the most abundant taxa and their composition at each site contribute to the distinct community compositions above and below the 70% serpentinized fluid threshold can be gained from the taxa-based dendrogram and the heatmap coloration. Note that taxa that cluster together in the dendrogram tend to be similarly distributed across sites. As an example, the anaerobic protist genus *Cyclidium*, and the heterotrophs *Ischnamoeba montana*, *Telaepolella* sp. Tib190, and *Hypotrichia* compose cluster 1 in Figure 4A. As shown by the coloration in the heatmap, these taxa are often the most abundant taxa at sites with compositions that are >70% serpentinized fluid and are often the least abundant at sites with <70% serpentinized fluid. As emphasized by the break along the rows of the heatmap, this cluster is distinct from another cluster containing only phototrophic and heterotrophic taxa. Within this larger cluster, there are two distinct subclusters designated 2 and 3 in Figure 4A. Cluster 2 is composed only of phototrophic protists, which are predominantly diatoms (Diatomea) with the addition of *Chlorophyceae* and *Spirogyra* sp. clade H CC 2012. Taxa within cluster 2 are often the most abundant at sites with compositions that are <70% serpentinized fluid. Cluster 3 is composed of both phototrophs and heterotrophs. Taxa within this cluster are never the most abundant at sites with <70% serpentinized fluid. The same is mainly true for sites with >70% serpentinized fluid as well, except for *Nautococcus solutus* and *Characium saccatum*, which are most abundant at sites with 70.1% (site 140110D) and 99.5% (site 140114V) serpentinized fluid, respectively. These taxa are green algae within the clade Chloroplastida. The dominance of Chloroplastida at these sites can be seen in Figure 2A. As mentioned above, site 140110D is centrally located along NMDS1 in Figure 3A.

Next to each taxonomic group on the right side of the heatmap in Figure 4A is the contribution of each taxonomic group (in%) to the Bray-Curtis dissimilarity between communities at sites with water compositions above and below 70% serpentinized fluid as determined by SIMPER analysis. As shown by the SIMPER values in Figure 4A, the taxonomic group with the greatest contribution (4.0%) to Bray-Curtis dissimilarity above and below 70% serpentinized fluid is the phototrophic class *Chlorophyceae*, and ASVs classified as *Chlorophyceae* are significantly more abundant at sites with <70% serpentinized fluid (Mann–Whitney U-test *p*-value < 0.05, see Table 3). The taxonomic group of Figure 4A with the second highest contribution to variation above and below 70% serpentinized fluid is the Ciliophora genus *Cyclidium*. Species of this genus are microaerophilic to anaerobic. *Cyclidium* is significantly more abundant at sites with compositions that are >70% serpentinized fluid (Mann–Whitney U-test *p*-value < 0.05, see Table 3). The third highest contribution is from the heterotrophic Amoebozoa species *Ischnamoeba montana*. ASVs classified as this species are significantly more abundant at sites with compositions that are >70% serpentinized fluid according to the Mann–Whitney U-test *p*-value < 0.05. Combined, the ASVs of the Diatomea taxa in Figure 4A contribute 12% to the observed variation, with ASVs classified as diatom taxa significantly more abundant at sites with <70% serpentinized fluid as shown by the Mann–Whitney U-test *p*-value < 0.05.

The dendrogram heatmap illustrates further evidence for a chemical ecotone. As mentioned above, there are two taxa that are phototrophs, a lifestyle that is generally more prevalent at sites with <70% serpentinized fluid, that occur at sites that fall centrally along NMDS1. In the dendrogram heatmap, one of these sites 140110D, is within a site-based cluster that includes site 140117F and site 140114V. Sites within this cluster also have a relatively high abundance of phylotypes classified as *Cyclidium*. As mentioned above, *Cyclidium* phylotypes are prevalent at sites with >70% serpentinized fluid. The co-occurrence of phototrophs (a lifestyle associated with sites that have <70% serpentinized fluid) and *Cylclidum* (phylotypes of which are more prevalent at sites with >70% serpentinized fluid) suggest these sites represent a transition between two community types and the dynamics of these sites, biological, physical and/or chemical, allow for these taxa to co-occur. Site 140117F is located in Shumayt where surrounding surface water and serpentinized fluid are geographically co-located and can mix. Shumayt provides an example of where mixing between fluids in one geographical location may result in a chemical ecotone. Sampling sites from Shumayt are indicated by black boxes in Figures 4A, B shows the composition of the most abundant taxa grouped by their lifestyles at these sites. At Shumayt, sites 140116D (14% serpentinized fluid) and 140116B (0% serpentinized fluid) are representative of the <70% serpentinized fluid community type, with both phototrophic and heterotrophic protists. Sites 140117G and 140117H (both 99.9% serpentinized fluid) have both anerobic and heterotrophic protists and are representative of the >70% serpentinized fluid community type. Site 140017F and 140117I at Shumayt do not fall distinctly within either community type, with >70% serpentinized fluid and the occurrence of phototrophs at site 140117I (centrally located in the NMDS ordination of Figure 3A) and the co-occurrence of anaerobic protists (*Cylclidium*) and phototrophs at site 140117F. Sites 140117F and 140117I may represent systems where intermittent fluid mixing is more likely to occur and therefore may allow two distinct community types to merge, resulting in a chemical ecotone.

Overall, the dendrogram heatmap of the most abundant taxa across sites shown in Figure 4A, reveals that sites with compositions that are >70% serpentinized fluid are dominated by heterotrophs and the anaerobic protist genus, *Cyclidium*. Sites composed of <70% serpentinized fluid are dominated by phototrophs and heterotrophs. Diatoms are particularly distinct in their contribution to the difference in protist community composition above and below 70% serpentinized fluid. Unlike diatoms, species of Chloroplastida appear capable of surviving sites with >70% serpentinized fluid. The two distinct community types that are defined by greater than or less than 70% serpentinized fluid may reflect the physiological properties of the most abundant taxa shown in Figure 4A. Physiological requirements may constrain protists to the specific geochemical and biological conditions we observe at our sampling sites which result from the process of serpentinization and subsequent mixing between serpentinized fluid and surrounding surface water. With that in mind, in the following section we turn our attention to geochemical and biological factors that change along the mixing gradient and that may influence the composition and richness of phototrophic, heterotrophic, and anaerobic protist communities.

### 4.3. Protist diversity as functions of lifestyle requirements

**FIGURE 5:**
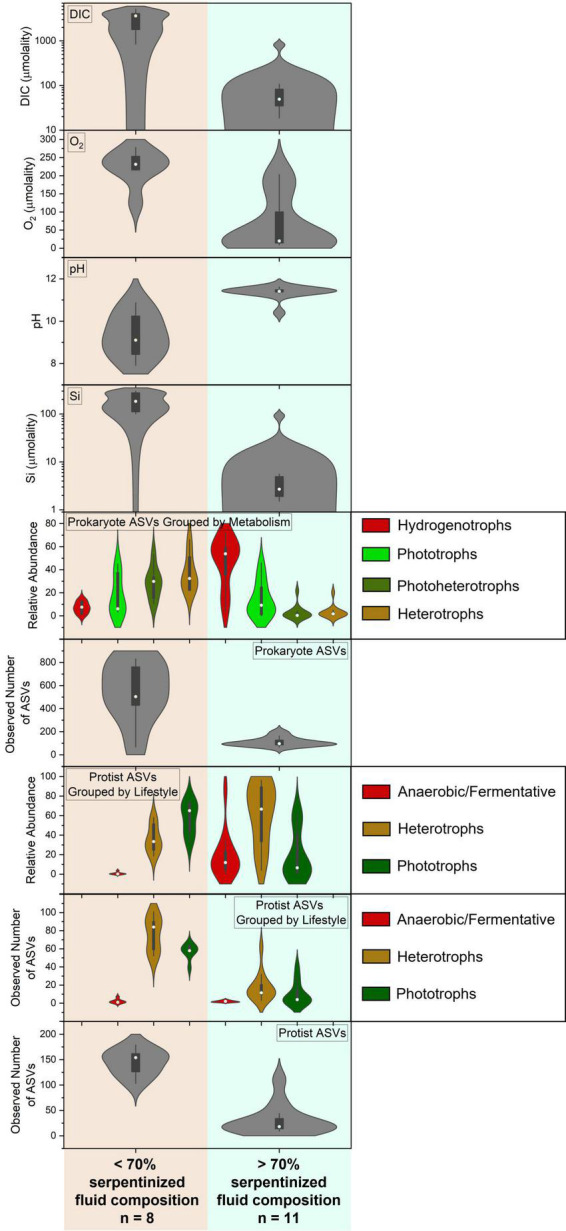
Summary of the variation in geochemical and microbial compositions of along the geochemical gradient of serpentinizing habitats in this study. Note the contrasts in protist diversity together with the geochemical and biological factors that correlate significantly with protist community composition and richness where water compositions are above and below 70% serpentinized fluid.

We investigated several geochemical and biological parameters, in addition to pH, that may contribute to the distinct transition in protist community composition and richness at 70% serpentinized fluid. The parameters include dissolved inorganic carbon (DIC), dissolved O_2_, the relative abundances of protists grouped by lifestyle, the richness of protists grouped by lifestyle, the relative abundances of prokaryotes grouped by metabolism type and the richness of prokaryote ASVs. Figure 5 contains a visual summary of the differences in these parameters above and below 70% serpentinized fluid composition. The violin plots in Figure 5 are kernel-fitted curves to the number of sites and their distribution across the range of data for each parameter. The larger the area under the curve, the higher the number of sites that fall within that data range. Within each violin plot is a box plot with the circle indicating the median, the ends of the box representing the 1*^st^* (25%) and 3*^rd^* (75%) quartiles of the data range, and the lines showing the total range of the data.

While there are many geochemical factors that are relevant to the survival of phototrophic protists, one that changes dramatically along the geochemical gradient is the availability of inorganic carbon, specifically CO_2_, which is fixed by phototrophs for incorporation into cell mass and respired for energy (Spalding, 1989). Dissolved inorganic carbon is significantly lower at sites where the water contains >70% serpentinized fluid, as illustrated by the DIC violin plot in Figure 5 and confirmed by the Mann–Whitney *p*-value < 0.05 (see Table 3). Violin plots in Figure 5 also reflect that the relative abundance and richness of phototrophic protists are also significantly lower at sites with >70% serpentinized fluid (Mann–Whitney *p*-value < 0.05, see Table 3).

**FIGURE 6:**
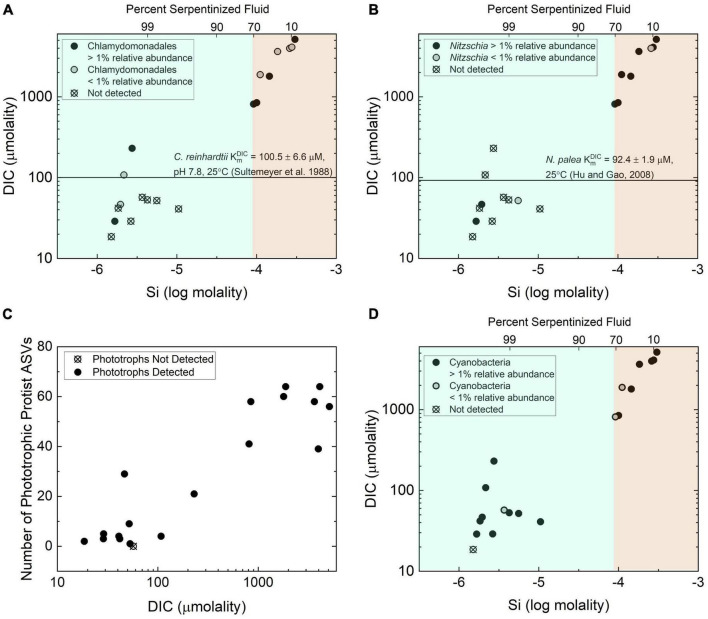
Distribution and richness of phototrophs with respect to inorganic carbon availability. **(A)** The distribution of protist phylotypes classified as belonging to the green algae order Chlamydomonadales as functions of total dissolved Si and DIC. The line shows the half saturation constant for DIC for photosynthetic activity by *Chlamydomas reinhardtii* reported in Sültemeyer et al. (1988). **(B)** The distribution of protist phylotypes classified as belonging to the diatom genus *Nitzschia*. The line is drawn to indicate the DIC half saturation constant for photosynthetic activity by *Nitzschia palea* reported in Hu and Gao (2008). **(C)** Richness quantified as the number of phototrophic protist ASVs, as a function of DIC. **(D)** The distribution of the prokaryote phylotypes classified as belonging to the phylum Cyanobacteria as functions of total dissolved Si and DIC.

Variable DIC abundances can limit the effectiveness of photosynthesis. As an example, the growth rate of the green algae *Chlamydomonas reinhardtii* depends on the availability of DIC (Colman et al., 2002; Yamano and Fukuzawa, 2009), and kinetic studies indicate that its half saturation constant (K_*m*_*^DIC^*), or concentration required to reach half the maximum photosynthetic rate, is 100.5 ± 6.6 μM DIC when adapted to high CO_2_ conditions. When adapted to low CO_2_ conditions, the half saturation constant is 21.9 ± 3.03 μM DIC (Sültemeyer et al., 1988). In a separate study, K_*m*_*^DIC^* for the diatom *Nitzschia palea* was determined to be 92.4 ± 1.99 μM when adapted to high CO_2_ conditions and 81.9 ± 4.7 μM when adapted to low CO_2_ conditions (Hu and Gao, 2008). As shown in Figures 6A, B except for three locations, sites with more than 70% serpentinized fluid have DIC concentrations that are lower than the K_*m*_*^DIC^* values for *C. reinhardtii* and *N. palea* adapted to high abundances of CO_2_. One of the exceptions, site 140115Y, is a site that occurs down an outflow from a spring of serpentinized fluid with elevated DIC obtained through reaction with the atmosphere. Site 140114V is another exception that is located in a region where surface water can mix with serpentinized fluid. And the third exception is site 140110D, which is a site where mixing between surface water and serpentinized fluid was visually observed at the time of sampling. Two of these exceptions, sites 140114V and 140110D, are a part of the site cluster in the dendrogram heatmap of Figure 4 that may reflect a chemical ecotone.

The influence the availability of DIC may have on protist diversity in serpentinizing systems is reflected in the distribution and diversity of phototrophic protist ASVs. As shown in Figure 6A, ASVs classified as belonging to the order Chlamydomonadales are predominantly absent from sites with >70% serpentinized fluid. The same is true of ASVs classified as belonging to the diatom genus *Nitzchia* as indicated in Figure 6B. A Mantel test shows that there is a significant positive correlation between Manhattan dissimilarity of log transformed DIC and Bray-Curtis dissimilarity of square-root transformed relative abundances of phototrophic protist ASVs, given the r-statistic = 0.44 and *p*-value < 0.005, which suggests the variation in phototrophic protist community composition is influenced by differences in DIC. As shown in Figure 6C, there is also a strongly significant correlation between DIC and phototroph richness, as indicated by the Pearson *r*-value = 0.92 and ANOVA *p*-value < 0.005.

The fact that DIC concentrations at most sites with >70% serpentinized fluid are lower than the known K_*m*_*^DIC^* for phototrophic microbial eukaryotes implies that these low concentrations are likely to limit photosynthetic activity and may explain the absence of Chlamydomonadales and *Nitzchia* ASVs at these sites. DIC correlation with richness suggests that an increased availability of inorganic carbon allows for more species to co-occur at sites with <70% serpentinized fluid and conversely, at sites with >70% serpentinized fluid, carbon limitation may enhance competitive interactions and therefore result in low phototrophic protist richness.

The correlation between the variation of phototrophic protist community composition and concentration of DIC may be explained by differences in carbon uptake and utilization between protist species. Phototrophic protists carry out photosynthesis with CO_2_ (Colman et al., 2002). Additionally, phototrophic protists, such as species of the green algae genus, *Chlamydomonas*, have been shown to take up HCO_3_^–^ and convert it to CO_2_ for use (Colman et al., 2002; Miura et al., 2004). This requires an adaptation generally referred to as a carbon concentrating mechanism that includes bicarbonate transporters and carbonic anhydrase for the reversible conversion of HCO_3_^–^ to CO_2_ (Colman et al., 2002). As shown in Supplementary Figure 5 the dominant aqueous species of inorganic carbon are CaCO_3_(aq) and CO_3_^–2^. Inorganic carbon species known to be used by phototrophs, CO_2_ and HCO_3_^–^, are far less abundant at sites where the water consists of >70% serpentinized fluid. While dissolved inorganic carbon is overall low and potentially limiting to phototrophic activity at sites with >70% serpentinized fluid, modeled chemical speciation of inorganic carbon along the gradient shows that CO_2_ is ∼6 orders of magnitude lower in abundance than HCO_3_^–^. While CO_2_ is more abundant at sites with <70% serpentinized fluid, the thermodynamic activity (≅ concentration) of CO_2_ is still ∼1 order of magnitude lower than that of HCO_3_^–^. Therefore, differences in phototrophic protist community composition may reflect the selection for organisms with carbon concentrating mechanisms (uptake and utilization of HCO_3_^–^), particularly at sites with >70% serpentinized fluid. Furthermore, serpentinizing systems may select for protists with novel adaptations that have yet to be observed in nature for taking up and using the more abundant forms of inorganic carbon, CO_3_^–2^ and aqueous CaCO_3_.

Interactions with bacterial phototrophs may be another factor influencing the diversity of phototrophic protists. There has long been a hypothesis that photosynthetic bacteria, namely Cyanobacteria, can outcompete green algae for inorganic carbon because they have more effective carbon concentrating mechanisms (Beardall and Raven, 2017). More recently, through culture-dependent competition studies, some green algae were shown to outcompete Cyanobacteria under CO_2_-poor conditions (Ji et al., 2017). The distribution along the geochemical gradient of 16S rRNA gene ASVs classified as belonging to the Cyanobacteria is shown in Figure 6D. At sites with compositions that are <70% serpentinized fluid, Cyanobacteria phylotypes co-occur with Chlamydomonadales and *Nitzschia* phylotypes. At sites with >70% serpentinized fluid, where Chlamydomonadales and *Nitzschia* phylotypes are generally not detected, Cyanobacteria phylotypes are. These differences in distribution suggests that at sites in this study where the water is composed of >70% serpentinized fluid Cyanobacteria may have more effective carbon concentrating mechanisms than organisms of the order Chlamydomonadales and the genus *Nitzschia*. At sites with <70% serpentinized fluid there is likely to be sufficient DIC to enable coexistence of these phototrophs. However, it is worth noting that some eukaryotic phototrophs were detected at sites with >70% serpentinized fluid as shown in the Figure 6C. Perhaps these microbial phototrophic eukaryotes have adaptations that allow them to co-occur with Cyanobacteria.

The diversity of diatoms, which are among the phototrophic protists we detected at our study sites, may be influenced by an additional geochemical factor unrelated to dissolved inorganic carbon. Many diatoms have an amorphous silica shell, therefore, SiO_2_(aq) (also referred to as orthosilicic acid, Si(OH)_4_) can be limiting to growth and survival (Martin-Jézéquel et al., 2000; Belton et al., 2012). Through the process of serpentinization, SiO_2_(aq), which is the dominant chemical species of Si in rain and circumneutral surface water, is removed as water infiltrates into the subsurface and reacts with rock, leaving serpentinized fluids relatively depleted in Si (Leong et al., 2021). While not all diatoms have silica shell, the availability of Si in serpentinized fluid may be a contributing factor to the overall diversity of diatoms in serpentinization-hosted ecosystems. The distribution and richness of diatom ASVs reflect the availability of Si with diatoms ASVs being present and most abundant as sites with <70% serpentinized fluid as shown in the NMDS plot of Supplementary Figure 8A and phylogenetic bar chart of Supplementary Figure 6A. At sites with >70% serpentinized fluid, diatoms are low in richness or absent as shown in Supplementary Figure 8B.

In the case of heterotrophic protists that may use O_2_ as an electron acceptor for respiration we hypothesize that the availability of O_2_ will influence the distribution and diversity. Heterotrophic protists have a wide range of O_2_ demands with some being microaerophiles (Scholander et al., 1952; Fenchel, 2012a). Given that O_2_ requirements vary among species of protists and that O_2_ gradients are known to shape heterotrophic protist communities (Fenchel, 1982; Fenchel et al., 1990; Fenchel and Finlay, 2008), it is possible that O_2_ abundance influences heterotrophic protist diversity along the geochemical gradient in the present study. As reflected in the O_2_ violin plot in Figure 5 sites with compositions that are >70% serpentinized fluid have significantly less dissolved O_2_ (average = 60.1 μ*m*, *n* = 12) than sites with <70% (average = 224.1 μ*m*, *n* = 7) (Whitney U-test *p*-value < 0.05, see Table 3). As indicated by the Mantel r-statistic = 0.53 and *p*-value < 0.005 for Manhattan dissimilarity of dissolved O_2_ concentration and Bray-Curtis dissimilarity of heterotrophic protist ASV square-root transformed relative abundances, there is a significant correlation between variations in O_2_ concentrations and heterotrophic protist community composition. Richness of heterotrophic protist ASVs also significantly correlates with dissolved O_2_, given the Pearson *r*-value = 0.77 and ANOVA *p*-value < 0.005. However, the abundance of heterotrophic protist is not significantly different between the two fluid types, which may reflect the potential for anaerobic respiration at sites with >70% serpentinized fluid.

In addition to O_2_ variability, another factor that may influence heterotrophic protist diversity is the composition of potential food sources. Predatory heterotrophic protists graze upon smaller organisms such as archaea, bacteria and even smaller protists (Straile, 1997; Pernthaler, 2005). In a stable isotope study following carbon flow from bacterial versus archaeal ammonia oxidizers to predatory protists in the ocean water column, some protist populations were shown to preferentially eat archaeal over bacterial ammonia oxidizers and *vice versa* (Salcher et al., 2016). Additional experimental studies show that grazing patterns of protists were shown to shape the composition of bacterial communities (Rønn et al., 2002). It is difficult to observe the direct correspondence between protist feeding patterns and prey community composition in the natural environment. To bridge this gap, there is a conceptual model in protist ecology proposed by Šimek et al. (2002) and reviewed in Pernthaler (2005) for how environmental context and protist grazing can shape bacterial (and potentially archaeal) communities. One extreme in this model is a scenario where environmental resources for prey populations are limited. In this scenario, protist grazing would have a large effect on prey survival and the environment likely selects for defense specialists. This is known as top-down control where predation drives community composition of prey. At the other extreme, bottom-up control, there are sufficient resources for prey and prey biomass is high, therefore prey community composition is driven by resource availability and the environment selects for competition specialists (Pernthaler, 2005). Putting our geochemical gradient in this context, prey populations in serpentinized fluids may fit within the top-down scenario given that as few as 1.16 × 10^5^ cells mL^–1^ were counted in a serpentinized fluid sample from a well (Fones et al., 2019). Surrounding surface water likely fits within the bottom-up scenario as high diversities of phototrophic ASVs, both bacterial and protist, imply these systems support relatively high productivity. Overall, with observed selectivity in protist grazing in other environments and the influence protist grazing may have in shaping prey communities, we expect that there will be correlations between heterotrophic protist community composition and the composition of potential food sources, archaea and bacteria, along this geochemical gradient.

**FIGURE 7:**
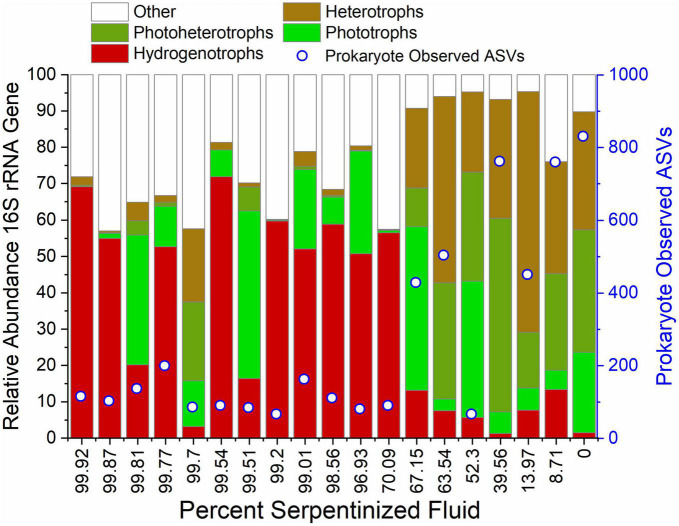
Diversity and inferred metabolic capacity of archea and bacteria in the Samail Ophiolite. 16S rRNA gene ASVs were taxonomically classified and grouped at the genus level and subsequently grouped by their potential metabolic capabilities. The bar-chart depicts only genera whose summed relative abundances at all sites is greater than 1%. Open circles indicate the number of 16S rRNA gene ASVs detected at each site.

We evaluated the composition of potential food sources for heterotrophic protists through 16S rRNA gene sequencing that captures both archaeal and bacterial (prokaryote) diversity and the resulting ASVs were classified using the SILVA database (Quast et al., 2013). 16S rRNA gene ASVs were grouped at the genus level, focusing on genera with average relative abundances across all sites >1%. Metabolisms were inferred based on 16S sequence homology with cultured archaea and bacteria, resulting in the list of taxa and assigned metabolisms in Supplementary Table 2, and the bar chart of taxa grouped by their metabolisms in Figure 7. As in the case of archaeal and bacterial metabolism types reported in Howells et al. (2022), there is a sharp transition in community composition at the point where water composition is ∼70% serpentinized fluid. As reflected in the prokaryote (archaea and bacteria) relative abundance violin plot of Figure 5, sites with <70% serpentinized fluid are dominated by phototrophs, photoheterotrophs and heterotrophs. Sites with >70% serpentinized fluid are dominated by organisms that can use H_2_ as an electron donor such as the aerobic hydrogen oxidizer genus, *Hydrogenophaga*, the hydrogenotophic methanogen, *Methanobacterium*, and sulfate reducers (Howells et al., 2022). A Mantel test between Bray-Curtis dissimilarity of square-root transformed prokaryote ASV relative abundances and Manhattan dissimilarity of log transformed Si concentration shows that there is a significant correlation between prokaryote ASV composition and the geochemical gradient, as indicated by the Mantel r-statistic = 0.70 and *p*-value < 0.005. Prokaryote communities at sites with <70% serpentinized fluid are significantly different from communities at sites with >70% serpentinized fluid, based on the ANOSIM r-statistic = 0.997 and *p*-value < 0.005. Prokaryote richness (number of ASVs) significantly correlates with log Si concentration (Pearson *r*-value = 0.84 and ANOVA *p*-value < 0.005) and richness is significantly higher at sites with <70% serpentinized fluid than at sites with >70% serpentinized fluid composition (Mann–Whitney U-test *p*-value < 0.05). For visualization of prokaryote community composition and richness changes along the gradient see Supplementary Figure 9.

A correlation between variations in potential food sources and heterotrophic protist community composition is revealed by a Mantel test between Bray-Curtis dissimilarity of square-root transformed relative abundances of prokaryote ASVs and heterotrophic protist dissimilarity. With a Mantel r-statistic of 0.71 and *p*-value < 0.005, there is a strong correlation between prokaryote and heterotrophic protist community composition dissimilarities. Pearson correlation analysis between the number of prokaryote and heterotrophic protist ASVs shows significant correlation between potential food source richness and heterotrophic protist richness, given the Pearson *r*-value = 0.85 and ANOVA *p*-value < 0.005. Overall, these correlations suggest that interactions among bacteria, archaea, and heterotrophic protists shape the microbial ecosystem dynamics along the geochemical gradient. It should be noted that both geochemical selectivity and the influence of predator-prey interactions can induce complex relations among the diversities of heterotrophic protists, bacteria, and archaea. The interactions themselves may be driven in part by the geochemical context, such as availability of oxygen. However, given the great interest in determining geochemical factors that shape bacterial and archaeal communities in serpentinization-hosted ecosystems, the observed correlations highlight the influence protist predation may have on shaping these communities.

The concentration of O_2_ may also influence the diversity of anaerobic protists detected in serpentinized fluids of Oman. Strict anaerobic protists carry out fermentation for energy and facultative protists can switch to fermentation when O_2_ is limited (Fenchel, 2012b). The exact cause of oxygen sensitivity in anaerobic protists is unknown, but it is hypothesized that anaerobic protists lack stress responses to O_2_ exposure, such as provided by the enzyme superoxide dismutase (Fenchel and Finlay, 1990). Additionally, in order to carry out fermentation anaerobic protists have an organelle called a hydrogenosome that is rich in O_2_-sensitive enzymes such as hydrogenases (Embley et al., 2003). In the present study, the most abundant anaerobic protist phylotype is classified as belonging to the genus *Cyclidium* (see Supplementary Figure 6C), whose members are known for their anaerobic capabilities. As an example, cultivation studies show that the species *Cyclidium porcatum* is a strict anaerobe (Clarke et al., 1993). Another species, *Cyclidium borrori*, isolated from a sulfur-rich microbial mat at Laguna Figueroa in Baja California, Mexico, can grow aerobically as well as under anaerobic, sulfide-rich conditions (Dyer, 1989). During anaerobic growth *C. borrori* mitochondria differentiate into smaller organelles (Dyer, 1989), which may be hydrogenosomes. Distribution studies of the species *Cyclidium citrullus* show that it lives in the oxyclines and anoxic zones of water-columns in Danish eutrophic fjords and is most abundant where O_2_ is no longer detectable (Fenchel et al., 1990). In addition, a characterization of ciliate communities down the water column of a eutrophic pond showed that *Cyclidium portucatum* exists at a water-column depth where the concentration of O_2_ is less than 50 μmolal (Guhl et al., 2006).

**FIGURE 8:**
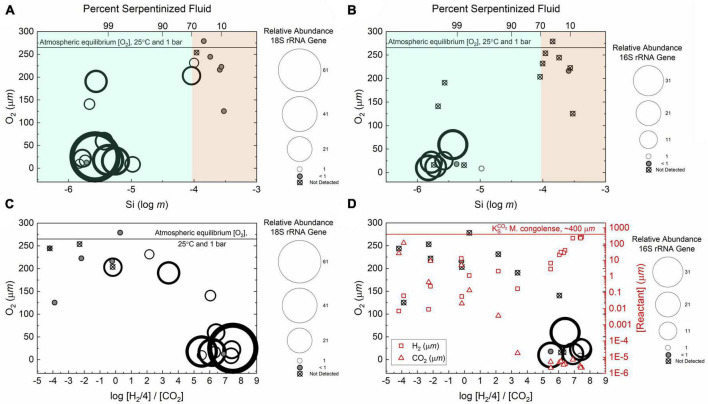
Relative abundances of anaerobic protists and the hydrogenotrophic methanogen, *Methanobacterium*. In these distribution plots the areas of the circles scale with the relative abundances of the 18S or 16S rRNA gene ASVs. **(A)** Relative abundances of the 18S rRNA gene ASVs classified as belonging to anaerobic protists as functions of dissolved O_2_ and Si. **(B)** Relative abundances of 16S rRNA gene ASVs classified as belonging to *Methanobacterium* as functions of dissolved O_2_ and Si. **(C)** Relative abundances of anaerobic protist ASVs as functions of dissolved O_2_ and the stoichiometrically balanced ratio of H_2_ and CO_2_ concentrations. **(D)** Relative abundances of *Methanobacterium* ASVs as in panel **(C)** with reactant concentrations: CO_2_ (triangles), H_2_ (squares). The line shows the CO_2_ half saturation constant for methanogenic activity by *Methanobacterium congolense* reported by Chen et al. (2019).

As discussed above, O_2_ varies along the geochemical gradient, being highest at sites with water compositions that are <70% serpentinized fluid that approach equilibrium with atmosphere as indicated by the horizontal line in Figure 8A. The relative abundances of protist taxa, indicated by circle size, that we identify as having potential anaerobic requirements or capabilities are plotted as functions of total dissolved Si and dissolved O_2_ in Figure 8A. Gray circles indicate sites where the relative abundance of fermentative protists is <1% and circles with an “x” correspond to sites where fermentative protists were not detected. The highest relative abundance of fermentative protist ASVs is at a site with 25 μmol O_2_. At the majority (86%) of sites where the water composition is <70% serpentinized fluid (*n* = 7), anaerobic protists are present at less than 1% relative abundance or are absent. In contrast, the relative abundance of anaerobic protists ASVs is >1% at the majority (92%) of sites (*n* = 12) with >70% serpentinized fluid. While anaerobic protists seem to prefer sites with >70% serpentinized fluid, where the concentration of O_2_ varies from 8.6 to 203.1 μmol, they are also detected in some of the sites with <70% serpentinized fluid where O_2_ is nearly at equilibrium with the atmosphere. There is one site where the water is composed of >70% serpentinized fluid that is also nearly in equilibrium with the atmosphere, which is the same outflow site (140115Y) that is relevant to the distribution of the phototroph taxa discussed above. Overall, the distribution pattern suggests that anaerobic protists have a selective advantage at sites where fluids are more influenced by the process of serpentinization and therefore relatively O_2_ limited.

Species of the genus *Cyclidium*, together with other anaerobic protist species, are known to have methanogen endosymbionts (Embley and Finlay, 1994; Esteban and Finlay, 1994). Methanogens are hypothesized to consume the fermentation product H_2_, which renders fermentation more energetically favorable and therefore makes the symbiotic relationship beneficial for the protist (Fenchel and Finlay, 1991). This connection is supported by the observation that less H_2_ evolves from protists with endosymbionts than free-living fermentative protists (Fenchel and Finlay, 1992). Endosymbiont methanogen species are within the orders Methanbacteriales, Methanosarcinales and Methanomicrobiales (Beinart et al., 2018). In our samples, the genus *Methanobacterium* (order Methanobacteriales) is most abundant at sites with water that is >70% serpentinized fluid (Howells et al., 2022) as indicated in Figure 8B, which shows the distribution of the *Methanobacterium* phylotype as functions of dissolved Si and O_2_. *Methanobacterium* phylotypes account for less than 1% of the relative abundance of ASVs or are absent from sites with water composed of <70% serpentinized fluid. *Methanobacterium* has greater relative abundance at many of the sites with >70% serpentinized fluid but is absent at sites with >∼60 μmol O_2_. While there is not complete correspondence with the distribution of the anaerobic protists, the relative abundance of *Methanobacterium* exceeds 1% at 5 of the 12 sites where the relative abundance of anaerobic protists also exceeds 1%. At these sites it is possible that *Methanobacterium* is living as an endosymbiont of anaerobic protists. In support of this hypothesis, the thermodynamic chemical activity of CO_2_ at sites with >70% serpentinized fluid is ∼8 orders of magnitude lower than the CO_2_ half saturation constant reported by Chen et al. (2019) for methane production by *Methanobacterium congolense* (see Figures 8C, D). This suggests that the concentration of CO_2_ in serpentinized fluids would be limiting to *M. congolense* activity and potentially other species of *Methanobacterium*. As CO_2_ is a fermentation product, an endosymbiotic relationship with anaerobic protists has the potential to circumvent CO_2_ limitation to species of *Methanobacterium* living in serpentinizing systems. At sites where *Methanobacterium* and *Cyclidium* phylotypes do not co-occur, it is possible that *Methanobacterium* strains are using formate as described by Fones et al. (2021).

Another advantage of the endosymbiotic relationship for methanogens is protection from an oxygenated atmosphere. A study on the response of the anaerobic protist *Metopus contortus*, which has methanogen endosymbionts, showed that methanogen viability within the protists was maintained upon prolonged exposure to O_2_ tensions up to 2% of atmospheric saturation (Fenchel and Finlay, 1990). The endosymbiotic methanogen maintained viability even after a 5-min exposure to 100% atmospheric saturation (Fenchel and Finlay, 1990). Finally, if anaerobic protists detected in this study are truly sensitive to O_2_, they are constrained to sites that are also relatively H_2_-rich as shown in Figures 8C, D. Having an endosymbiotic relationship with an H_2_ consumer is therefore beneficial for a protist carrying out fermentation as the endosymbiont can lower the internal concentration of H_2_ making the metabolism more favorable.

Overall, while not conclusive, the co-occurrence of anaerobic protists and *Methanobacterium* highlights the potential for serpentinization-hosted ecosystems to support complex biological interactions. An additional factor that may contribute to the dominance of *Cyclidium* is the potential that organisms of this genus are carrying out fermentation (substrate level phosphorylation) and not oxidative phosphorylation, thereby bypassing the proton requirement for ATP synthesis. Fermentative protist may therefore have the advantage at sites with >70% serpentinized fluid, which also all have pH > 10.

## 5. Significance of protist diversity in serpentinizing systems

Correlations between heterotroph protist diversity and prokaryote diversity are observed in this study and the distribution of potential protist endosymbionts corresponds to anaerobic protist distribution. Looking at the data this way compels one to think that prokaryote diversity may drive protist diversity and *vice versa*. Indeed, correlation analysis between dissimilarity of all protist ASVs and dissimilarity of prokaryote ASVs reveals that overall protist community composition strongly and significantly correlates with prokaryote community composition, given the Mantel r-statistic = 0.73 and *p*-value < 0.005. Richness of protist and prokaryote ASVs are also strongly correlated, indicated by the Pearson *r*-value = 0.90 and ANOVA *p*-value < 0.005. While this may be due in part to predator-prey interactions and endosymbiosis, it may also reflect the selective nature of serpentinized fluid. As the contribution from serpentinized fluid increases, community compositions shift and less diversity is supported. Although there are complexities in disentangling the extent to which geochemical processes or biological interactions are driving protist diversity, the serpentinization gradient highlights the possibility that the environment may select for close associations between protists and prokaryotes. Furthermore, the process of serpentinization is thought to be ubiquitous in the solar system and may support life elsewhere such as the icy satellites of Jupiter and Saturn, Europa and Enceladus (McCollom, 1999; Glein et al., 2008; Waite et al., 2017). The potential for close associations between protists and bacteria and archaea in serpentinized fluids on Earth suggest that these satellites may support more complex biological interactions and complex life than originally anticipated.

With increased focus on the application of serpentinizing systems for energy, specifically H_2_ production, and carbon sequestration, acknowledging the diversity of protists in these systems is key to understanding the efficacy of serpentinization for these applications. The presence of phototrophic protists is encouraging as photosynthetic activity may enhance CO_2_ sequestration from the atmosphere. However, as discussed above, the greater the contribution of serpentinized fluid to the water composition, the lower the occurrence and richness of phototrophs. Given that the concentration of CO_2_ at sites with >70% serpentinized fluid may potentially limit photosynthetic activity by protists, CO_2_ limitation in sequestration efforts may be overcome by injections of CO_2_ that increase the concentration to a point above protist requirements for photosynthesis. On the other hand, heterotrophic protists and protists that live in association with methanogens may enhance carbon release to the atmosphere and may render serpentinizing systems non-viable for carbon sequestration. Serpentinized fluid outflow channels may be a good target for carbon sequestration as O_2_ from the atmosphere can limit methanogen growth and CO_2_ from the atmosphere may allow organisms like *Chlamydomas reinhardtii* to survive as shown by investigations of site 140115Y in this study. Moving forward, now that the presence of protists in serpentinizing systems has been observed through 18S rRNA gene sequencing, we can consider their involvement in carbon cycling in these systems and give thought to what their presence in serpentinizing systems on Earth means for possibility of life elsewhere.

## Data availability statement

The datasets presented in this study can be found in online repositories. The names of the repository/repositories and accession number(s) can be found below: https://www.ncbi.nlm.nih.gov/, PRJNA919024.

## Author contributions

The lab of GG at Arizona State University provided the supplies and the protocol for assembling the 18S rRNA gene amplicon sequencing library. FD helped AH to assemble the sequencing library. AH conducted the sequencing data processing, analysis, and interpretation with the advisement of FD and GG. AH conducted the geochemical sampling and analysis with the lab of ES at Arizona State University. AH conducted the aqueous chemical modeling with the advisement of ES. AH conducted the interpretation of sequencing and geochemical data and the write up of the results with the advisement of ES, GG, and FD. All authors contributed to the article and approved the submitted version.
