# Supplementary material for: An examination of protist diversity in serpentinization-hosted ecosystems of the Samail Ophiolite of Oman
Source: Front Microbiol. 2023 May 4;14:1139333. doi: 10.3389/fmicb.2023.1139333 (PMC10192764; doi:10.3389/fmicb.2023.1139333)
Supplement: Supplementary file 2 [file Data_Sheet_1.docx]

Supplementary Material


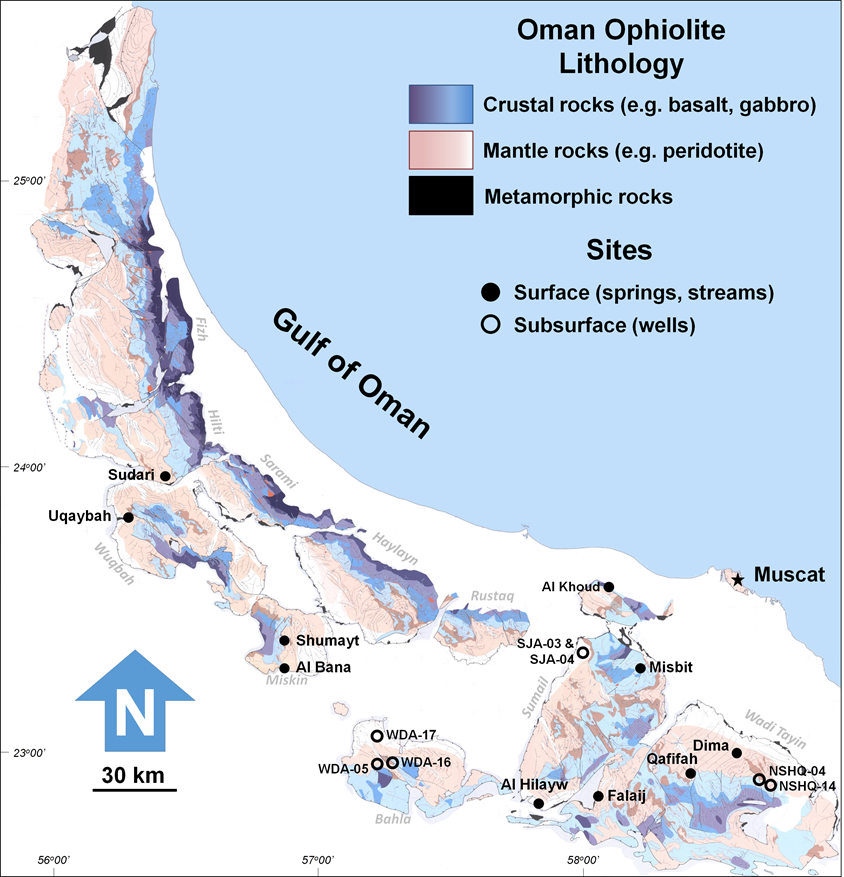


**Supplementary Figure 1. Geologic map of the Samail Ophiolite in Oman with locations of the study sites.** Modified from Howells et al. (2022).


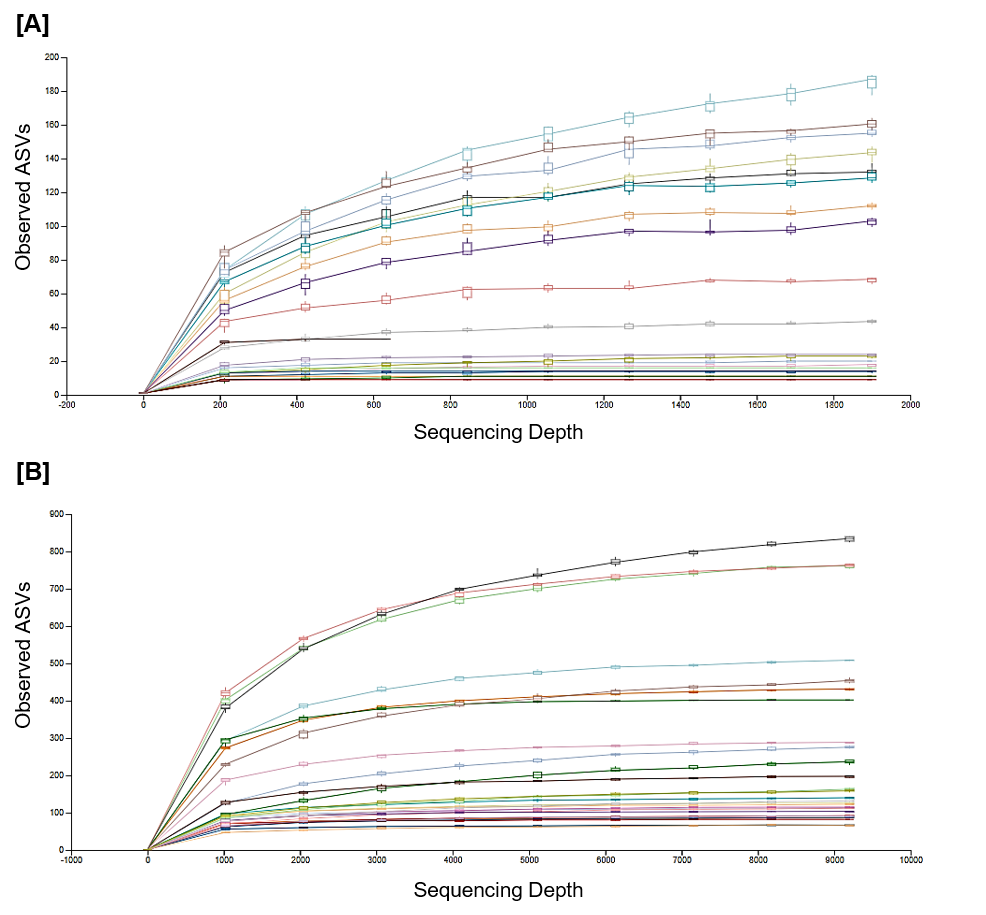


**Supplementary Figure 2. Rarefaction curves of protist and 16S ASVs.** **[A]** Curves of protist ASVs for each site rarefied to 1900. **[B]** Curves of 16S ASVs rarefied to 9200. Overall, more 16S ASVs were detected than 18S sequences.


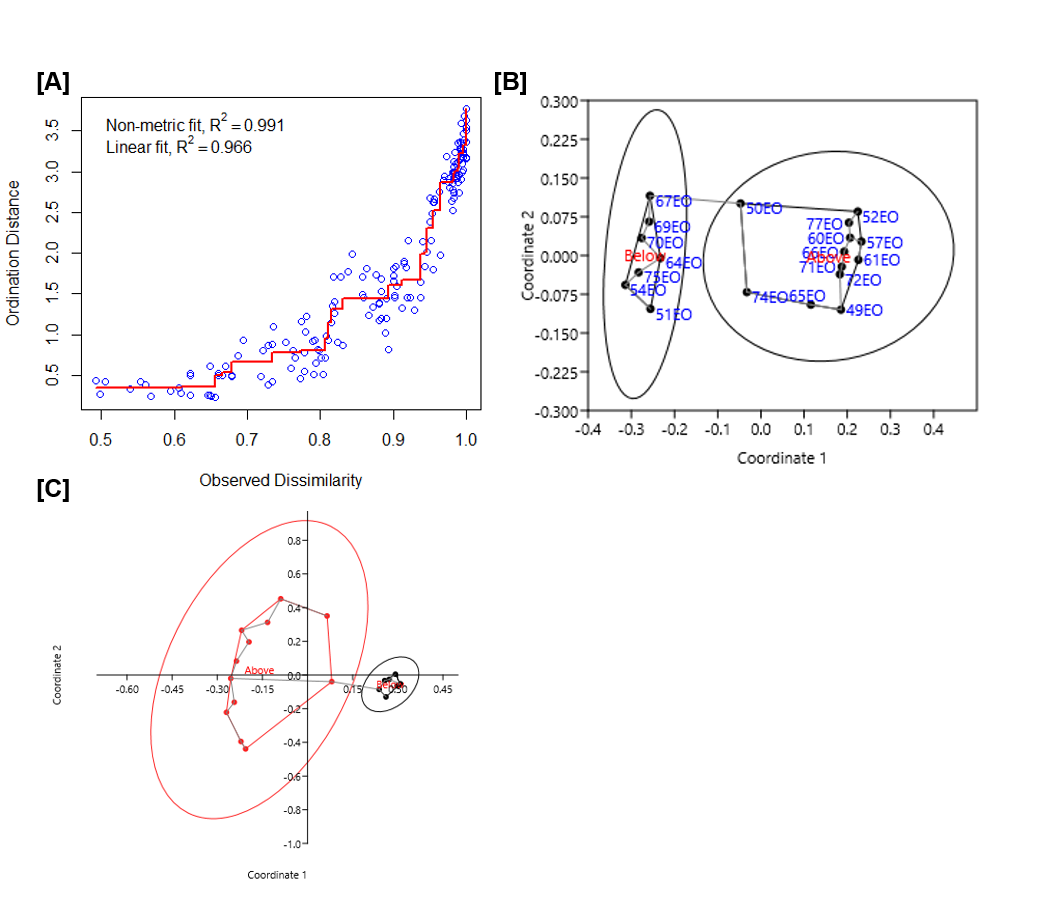


**Supplementary Figure 3. Supplementary material for NMDS ordination of Bray-Curtis dissimilarity of rarefied, square-root transformed protist ASV relative abundances. [A]** Stress plot of the Euclidean dissimilarity analysis of the ordination distances versus the observed Bray-Curtis dissimilarity. The r-squared of 0.966 for the linear fit suggests the two-dimensional ordination sufficiently represents community variation between sites. **[B]** NMDS plot, made using Past 4.02, with 95% concentration ellipses added. **[C]** Principal coordinates analysis (PCO) also made in Past 4.02 with 95% concentration ellipses added.


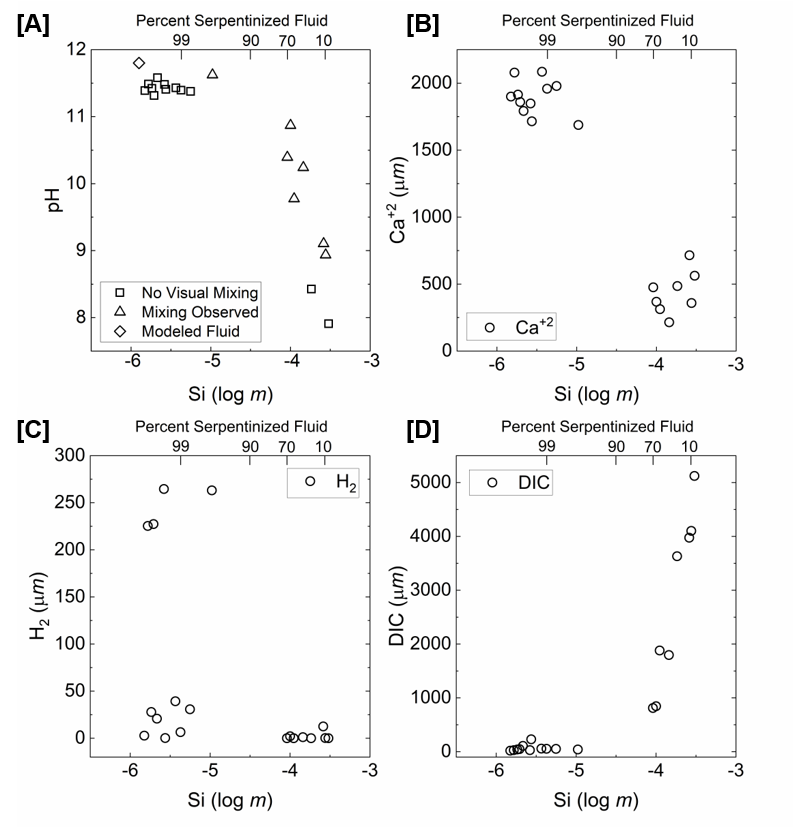


**Supplementary Figure 4. Geochemical parameters directly influenced by the process of serpentinization.** Total dissolved Si is used as an indicator of the extent of mixing between serpentinized fluid and surrounding surface water. The diamond in **[A]** shows the pH and Si concentration of modeled serpentinized endmember fluid described in Leong et al. (2021). The mixing model between the predicted Si concentration of the serpentinized fluid end member and the highest measured Si concentration was used to calculate percentages of endmembers in fluid mixtures as shown on the top abscissa. Triangles in **[A]** indicate study sites where mixing was observed. Squares are sites where mixing was not observed. The concentrations of Ca^+2^, H_2_ and DIC are shown in **[B]**, **[C],** and **[D]**, respectively.

**Supplementary Table 1. Modeled inorganic carbon species activity and affinity of amorphous silica and quartz formation.** Activity is approximately equal to molality. Affinity is in kcal per mol reaction.

| Site ID | Modeled dissolved inorganic carbon species activity | | | | | Affinity of SiO_2_ mineral formation | |
| --- | --- | --- | --- | --- | --- | --- | --- |
|  | CO_2_ | HCO_3_^-^ | Ca(HCO_3_)^+^ | CO_3_^-2^ | Ca(CO_3_) | Amorphous  SiO_2_ | Quartz |
| 140115Y | 4.65E-12 | 1.30E-06 | 1.40E-08 | 4.90E-05 | 5.76E-05 | -7.13 | -5.37 |
| 140114V | 1.75E-11 | 3.68E-06 | 3.95E-08 | 9.99E-05 | 1.27E-04 | -6.63 | -4.87 |
| 140114T | 3.33E-12 | 6.36E-07 | 8.07E-09 | 1.60E-05 | 2.52E-05 | -7.01 | -5.27 |
| 140113O | 3.78E-12 | 7.72E-07 | 1.03E-08 | 2.13E-05 | 3.50E-05 | -6.70 | -4.96 |
| 140111G | 8.73E-06 | 0.00364 | 8.10E-06 | 1.87E-04 | 5.12E-05 | -1.21 | 0.56 |
| 140111H | 1.34E-07 | 8.35E-04 | 8.68E-07 | 6.24E-04 | 7.81E-05 | -2.51 | -0.73 |
| 140111I | 3.60E-09 | 1.21E-04 | 2.37E-07 | 4.81E-04 | 1.10E-04 | -3.47 | -1.68 |
| 140111F | 2.79E-12 | 6.06E-07 | 7.08E-09 | 1.67E-05 | 2.37E-05 | -6.04 | -4.28 |
| 140116B | 1.16E-04 | 0.00483 | 1.80E-05 | 2.62E-05 | 1.23E-05 | -1.11 | 0.64 |
| 140116D | 4.92E-06 | 0.00324 | 1.29E-05 | 2.76E-04 | 1.42E-04 | -1.42 | 0.33 |
| 140117I | 6.99E-12 | 9.21E-07 | 1.18E-08 | 1.65E-05 | 2.92E-05 | -7.04 | -5.31 |
| 140117G | 2.17E-12 | 3.27E-07 | 4.27E-09 | 6.60E-06 | 1.16E-05 | -7.22 | -5.48 |
| 140117F | 2.20E-12 | 4.32E-07 | 5.46E-09 | 1.10E-05 | 1.74E-05 | -6.79 | -5.04 |
| 140117H | 2.21E-12 | 4.08E-07 | 5.67E-09 | 1.01E-05 | 1.82E-05 | -7.26 | -5.53 |
| 140110B | 2.64E-05 | 0.00343 | 1.16E-05 | 5.50E-05 | 2.34E-05 | -1.37 | 0.39 |
| 140112L | 4.28E-07 | 0.0012 | 2.05E-06 | 4.08E-04 | 8.51E-05 | -2.22 | -0.45 |
| 140110D | 2.06E-08 | 2.43E-04 | 6.55E-07 | 3.51E-04 | 1.15E-04 | -3.05 | -1.28 |
| 140112M | 5.40E-12 | 8.83E-07 | 1.16E-08 | 1.92E-05 | 3.17E-05 | -6.33 | -4.59 |
| 140110C | 5.11E-12 | 8.81E-07 | 1.14E-08 | 2.01E-05 | 3.20E-05 | -6.46 | -4.71 |





**Supplementary Figure 5.** Modeled chemical activities of inorganic carbon species plotted against total dissolved Si concentrations, which reflects the contribution of serpentinized fluid. The red lines are the DIC half saturation constants (K_m_^DIC^) for photosynthetic activity of *C. reinhardtii* (Sultenmeyer et al. 1988) and *N. palea* (Hu and Gao, 2008).


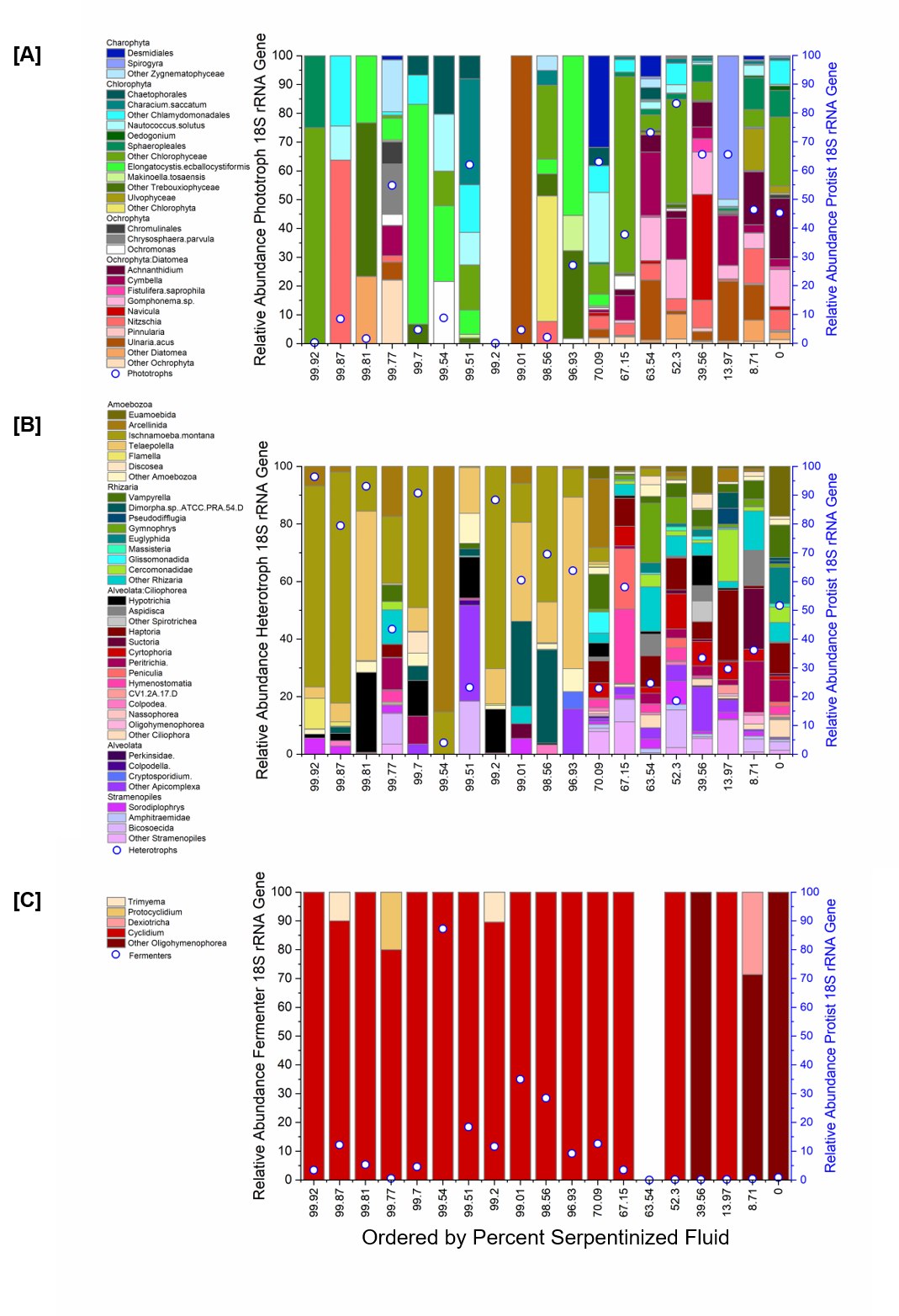


**Supplementary Figure 6. Diversity of protist taxa grouped by their lifestyle.** Each chart shows taxa with the given metabolism and the relative abundance of those taxa within the respective metabolism phylotypes. The blue circles are relative abundances of metabolism phylotypes within the whole protist community. **[A]** Photosynthetic protist taxa, **[B]** heterotrophic protist taxa and **[C]** anaerobic/fermentative protist taxa.





**Supplementary Figure 7. Supplementary material to most abundant protist taxa heatmap dendrogram.** Bars filled with diagonal lines show the summed relative abundance of SILVA 32 level 9 taxa whose average relative abundance across sites is > 1%. These taxa were used to construct the heatmap dendrogram shown in **Figure 6**.


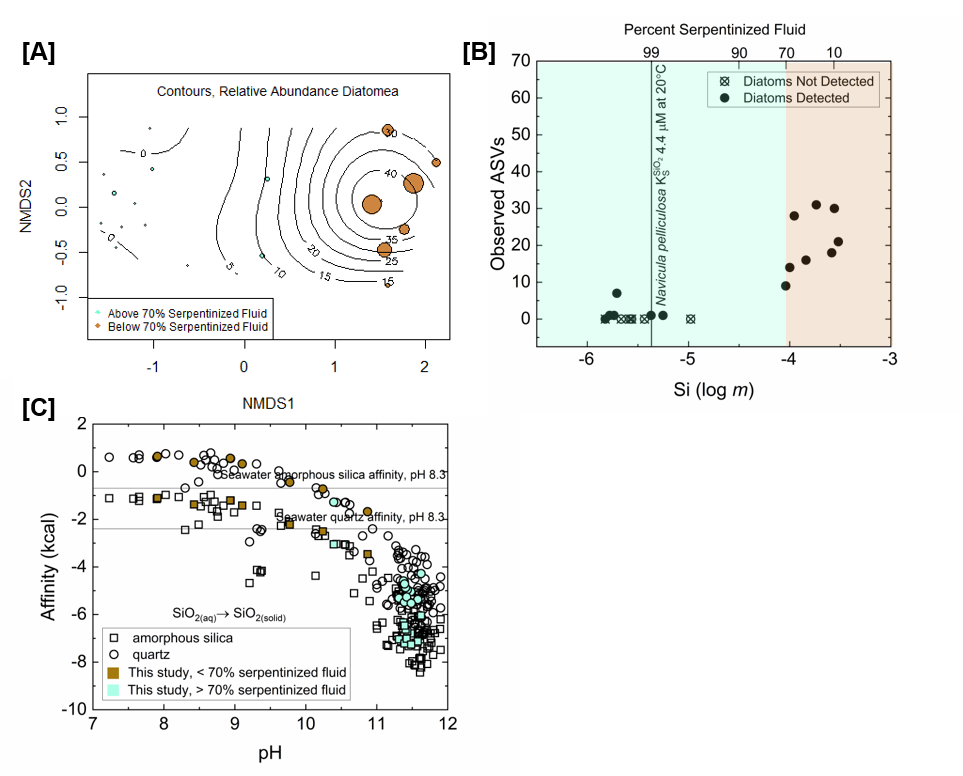


**Supplementary Figure 8. Distributions and richness of diatoms with respect to Si availability and silica mineral chemical affinities. [A]** The relative abundance of diatom phylotypes contoured onto the NMDS plot of **Figure 3A. [B]** The number of ASVs classified as diatoms as a function of total dissolved Si. **[C]** The affinity for the formation of amorphous silica and quartz as functions of pH. Open symbols show data from serpentinized fluids in Oman reported in Leong (2020) and include data from sites in this study (filled symbols).


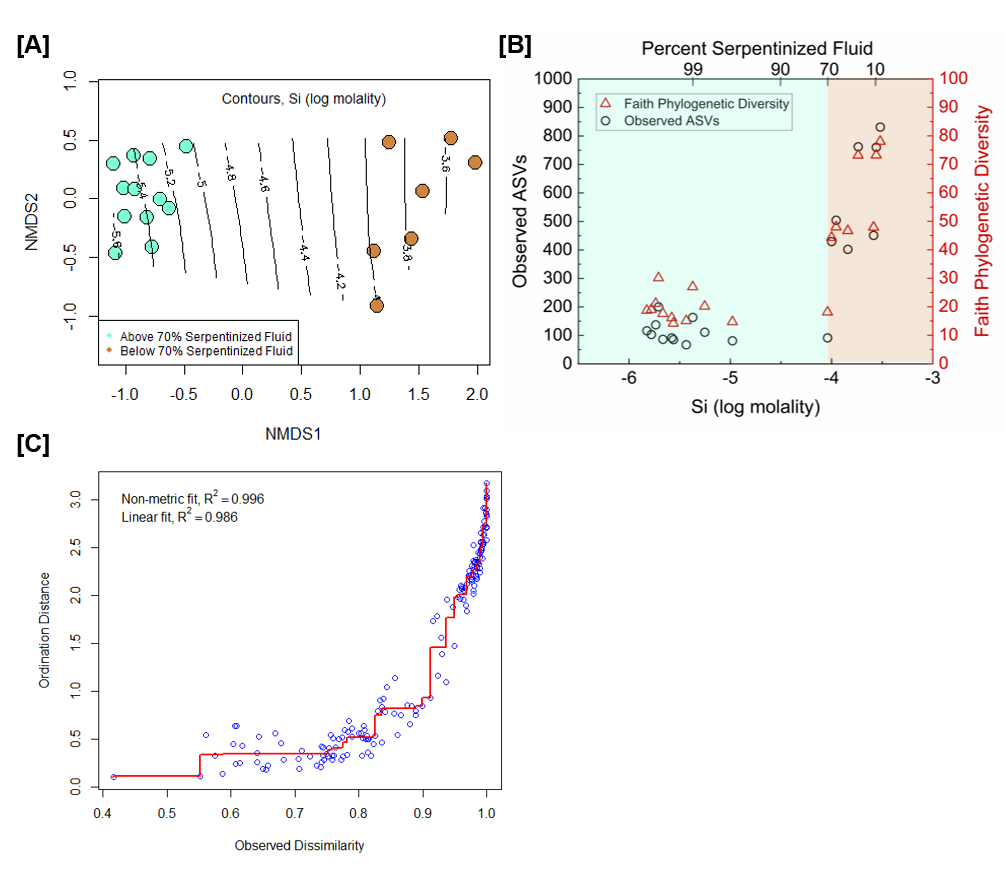


**Supplementary Figure 9. Analaysis of prokaryote diversity through 16S rRNA gene sequencing. [A]** NMDS ordination of Bray-Curtis dissimiarlity of rarefied, square-root transformed 16S rRNA gene ASV relative abundances. The stress is 0.07. Contours are fit to total dissolved Si using a maximum liklihood method. The color of the symbols correpond to above or below 70% serpentinized fluid. **[B]** The richness (observed number) of rarefied 16S rRNA gene ASVs, together with the Faith phylogenetic diversity of 16S ASVs. **[C]** Stress plot for the NMDS ordination of the 16S ASV Bray-Curtis dissimilarity.

**Supplementary Table 2. Prokaryote taxa relative abundances and assigned metabolisms.** See attached excel file.
